# Probing the DNA Reactivity and the Anticancer Properties of a Novel Tubercidin-Pt(II) Complex

**DOI:** 10.3390/pharmaceutics12070627

**Published:** 2020-07-04

**Authors:** Stefano D’Errico, Andrea Patrizia Falanga, Domenica Capasso, Sonia Di Gaetano, Maria Marzano, Monica Terracciano, Giovanni Nicola Roviello, Gennaro Piccialli, Giorgia Oliviero, Nicola Borbone

**Affiliations:** 1 CESTEV, University of Naples Federico II, via Tommaso De Amicis, 95, 80145 Napoli, Italy; stefano.derrico@unina.it (S.D.); domenica.capasso@unina.it (D.C.); 2 Dipartimento di Medicina Molecolare e Biotecnologie Mediche, University of Naples Federico II, via Sergio Pansini, 5, 80131 Napoli, Italy; andreapatrizia.falanga@unina.it; 3 Istituto di Biostrutture e Bioimmagini, CNR, via Mezzocannone, 16, 80134 Napoli, Italy; digaetan@unina.it (S.D.G.); giroviel@unina.it (G.N.R.); 4 Dipartimento di Farmacia, University of Naples Federico II, via Domenico Montesano, 49, 80131 Napoli, Italy; maria.marzano@unina.it (M.M.); monica.terracciano@unina.it (M.T.); picciall@unina.it (G.P.); nicola.borbone@unina.it (N.B.); 5 ISBE-IT—Candidate National Node of Italy for ISBE, Research Infrastructure for Systems Biology Europe, University of Naples Federico II, via Domenico Montesano, 49, 80131 Napoli, Italy

**Keywords:** tubercidin, cisplatin, platinum complexes, nucleosides, cancer, CD spectroscopy, DNA, cell viability assay, apoptosis, ESI-MS stability studies

## Abstract

Herein, we reported on the synthesis of a novel Pt(II) neutral complex having as ligand the nucleoside tubercidin, a potent anti-tumor agent extracted from the bacterium *Streptomyces Tubercidicus*. In detail, the chelation of the metal by a diamine linker installed at C6 purine position of tubercidin assured the introduction of a cisplatin-like unit in the molecular scaffold. The behavior of the synthesized complex with a double-strand DNA model was monitored by CD spectroscopy and compared with that of cisplatin and tubercidin. In addition, the cell viability was evaluated against HeLa, A375 and WM266 human cancer cell lines using the MTT test. Lastly, the results of the apoptotic assay (FITC Annexin V) performed on the HeLa cancer cell line are also reported.

## 1. Introduction

**Figure 1:**
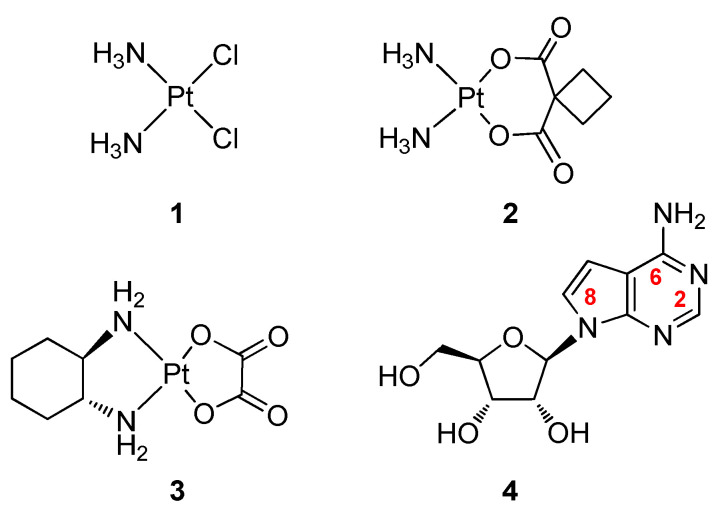
The structures of cisplatin (**1**), carboplatin (**2**) oxaliplatin (**3**) and tubercidin (**4**).

Cancer represents the second most frequent cause of death worldwide, particularly in rich countries where it reaps victims just like cardiovascular diseases. Considering that cardiovascular diseases are decreasing thanks to the availability of better treatments and prevention, cancer mortality will most likely become the leading cause of death for adults of middle age in the future. Despite the huge progress achieved in the field of molecular biology with targeted therapies, chemotherapy remains the most effective weapon in the presence of inoperable tumors or metastases [1]. Metallodrugs are a class of chemotherapeutics introduced in the market to improve the life expectancy of cancer patients [2]. The progenitor of such compounds is cisplatin (**1**, Figure 1) [3], which was approved by the Food and Drug Administration (FDA) in 1978 for the treatment of ovarian and testicular cancers. Cisplatin has a unique mode of action that consists of binding the nuclear DNA of cancer cells, inducing, in the final stage, apoptosis [4]. The severe drawbacks of cisplatin and the acquired resistance of many tumors often force patients to change therapy during the drug regimen. Only a few platinated compounds have been approved by the FDA for the treatment of cancers [5,6,7]; among them, carboplatin (**2**) and oxaliplatin (**3**) are characterized by a better tolerated toxicity profile than cisplatin, thanks to the presence of the bidentate dicarboxylate leaving groups, which modulate the reactivity at the metal center [8]. Notwithstanding this favorable achievement, they cannot be considered ideal drugs because of the frequent onset of drug resistance during treatment.

**Figure 2:**
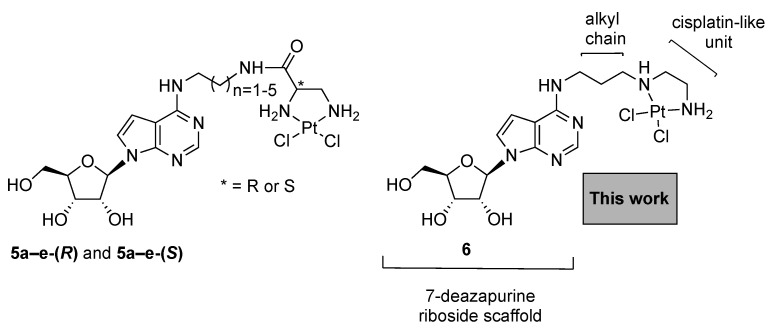
The structures of platinated nucleoside complexes **5a–e-(*R*)**, **5a–e-(*S*)** and **6** carrying the 7-deazapurine riboside scaffold.

Numerous classes of Pt(II) compounds have been obtained starting from cisplatin by introducing in the place of the ammonia ligands lipids [9,10,11], peptides [12,13], sugars [14,15,16], natural products [17], and some of them have shown interesting anti-proliferative effects. Considering that many anticancer and antiviral drugs currently approved are nucleoside/nucleotide-based [18,19,20,21], the study of the ligand properties of modified nucleosides and nucleotides towards Pt(II) metal centers is an interesting field in the design of novel anti-neoplastic agents [22,23,24,25,26,27,28,29]. In addition, the involvement of protein transporters located on cellular membranes in the molecular recognition and consequent internalization of nucleosides and nucleotides makes them even more attractive for the design of novel metal-based chemotherapeutics [30]. The nucleoside 7-deazapurine riboside, namely tubercidin (**4**), is a potent antibiotic and anti-tumor agent that is phosphorylated by cellular kinases to the corresponding triphosphate, and, once incorporated into DNA or RNA, could induce damage to nucleic acid functions [31]. In addition, tubercidin takes part in several cellular processes, such as pre-mRNA processing, mitochondrial respiration and purine synthesis [32]. In the last few years, many synthetic efforts have been devoted to the preparation of tubercidin analogues, with the aim to reduce its strong toxicity. Therefore, the molecular scaffold of tubercidin has been altered, by introducing modifications mainly at the C6, C7 and C8 purine positions. Interestingly, the substitution at the C6 purine position with alkyl, aryl and heteroaryl substituents has generated analogues endowed with significant biological activities [32]. Among all the tubercidin analogues synthesized so far, the C6 alkylamino derivatives are unprecedented. For the potential biological implications, the presence of two adjacent amine functions on the same alkyl chain is more appealing, as the nucleophilicity of the two nitrogen atoms may be exploited to chelate important bio-metals [33]. Accordingly, we probed the anticancer potential of some platinum(II) complexes carrying the cisplatin-like moiety linked to tubercidin through a *N*-alkyl-amide diamino spacer installed at the C6 purine position (**5a–e-(*R*)** and **5a–e-(*S*)**, Figure 2) [34,35].

The lack of the purine N7 atom assured the obtainment of neutral Pt(II) complexes, as a consequence of the sole metalation of the pendant diamine moieties. The compounds **5a–e** were screened on two human cancer cell lines and their capability to react with the guanosine 5′-monophosphate, the shortest DNA fragment, was assessed through ^1^H-NMR spectroscopy. To assess the effect of a simplified molecular scaffold on the reactivity toward DNA, herein we report on the synthesis of the novel complex **6**, obtained by directly connecting the tubercidin scaffold to the cisplatin-like unit [36,37,38]. The complex **6** was tested for its capability to react with a model DNA duplex and the results were compared with those obtained from cisplatin and an equimolar mixture of representative complexes **5b-(*R*)*/*5b-(*S*)** (*n* = 2, from now **5b**). Furthermore, the preliminary in vitro cytotoxic activity of the complex **6** is also reported. We anticipate that the complex **6** reacted more quickly than both cisplatin and the previously synthesized complex **5b** towards the DNA duplex, by inducing conformational distortions to the double helix like those elicited by cisplatin.

## 2. Materials and Methods

### 2.1. General Methods

We obtained all solvents and reagents from commercial sources and used them in the chemical synthesis of the herein-described derivatives without further purification steps. We acquired the ^1^H- and ^13^C-NMR spectra on a Bruker Avance Neo 400 MHz instrument (Bruker-Biospin, Billerica, MA, USA) using the deuterated solvents CD_3_OD, (CD_3_)_2_SO and CDCl_3_. The chemical shifts, given in parts per million (δ) and referenced to the residual solvent signal (^1^H: 3.31 CD_2_HOD, 2.54 (CD_3_)(CD_2_H)SO, 7.27 CHCl_3_; ^13^C: 49.0 CD_3_OD, 40.4 (CD_3_)_2_SO, 77.0 CDCl_3_), were assigned by 2D-NMR analysis. All acquired NMR spectra were processed by the iNMR software (Nucleomatica, Molfetta, Italy). The UV spectra were measured using a Jasco V-530 UV spectrophotometer (Jasco Europe, Cremella, Italy). The IR spectra were registered on a Jasco FT-IR 430 spectrophotometer (Jasco Europe, Cremella, Italy). The ESI-MS spectra were recorded on an Applied Biosystem mass spectrometer equipped with a triple quadrupole mass analyzer (ThermoFisher, Waltham, MA, USA) and on an LTQ-XL mass spectrometer equipped with an ion-trap (ThermoScientific, Waltham, MA, USA). The HRESI–MS spectra were obtained with a Thermo Orbitrap XL mass spectrometer (Thermo Fisher Scientific, Waltham, MA, USA). Chromatographic separations were performed by using silica gel 60, 70–230 mesh (Merck, Darmstadt, Germany), while the TLC analyses were carried out using 0.2 mm thick F254 silica gel plates (Merck, Darmstadt, Germany), with TLC spot visualization achieved under UV light (254 nm). The anionic exchange chromatography was performed on a glass column (10 mm diameter, 100 mm length) with a fused-in sintered glass-disc (bore of plug 2.5 mm) containing an OH^–^ form resin, 200–400 mesh, loading = 1.2 mmol/mL (Dowex^®^ 1X8, Sigma-Aldrich, Milan, Italy). The high-performance liquid chromatography (HPLC) was performed on an UP-2075 Plus pump equipped with a UV-2075 Plus UV detector (Jasco, Cremella, Italy). The ODNs’ HPLC purifications were performed on a Nucleogel^®^ SAX 1000-8 strong anion exchange column (Macherey-Nagel, Duren, Germany) using the following conditions: buffer A, 20 mM NaH_2_PO_4_ aqueous solution (pH 7.0) containing 20% (*v/v*) CH_3_CN; buffer B, 1 M NaCl, 20 mM NaH_2_PO_4_ aqueous solution (pH 7.0) containing 20% (*v/v*) CH_3_CN; linear gradient from 0% to 100% B in 30 min, flow rate of 1.2 mL/min. The purified ODNs were desalted by molecular exclusion chromatography on Biogel P2 fine (Biorad, Milano, Italy). The HPLC stability experiments were performed using a 5 μm, 250-10 C-18 reverse-phase column (Purosphere^®^ STAR, Merck, Darmstadt, Germany) eluted with a linear gradient of MeOH in H_2_O (0–100%) with a flow rate of 2.0 mL/min.

Human adenocarcinoma (HeLa), human low metastatic melanoma (A375) and human metastatic melanoma (WM266) cell lines were from ATCC (Manassas, VA, USA); normal human dermal fibroblasts (HDF) were from Arterra Biosciences (Napoli, Italy). HeLa, A375 and HDF were grown in DMEM medium supplemented with fetal bovine serum (FBS, 10%), glutamine (1%), penicillin (100 U/mL) and streptomycin (100 µg/mL, Euroclone, Milano, Italy). WM266 were grown in RPMI supplemented with FBS (10%), glutamine (1%), penicillin (100 U/mL) and streptomycin (100 µg/mL) [39]. Cells were maintained at 37 °C in 5% CO_2_ in humidified air. 

### 2.2. Chemistry

#### 2.2.1. Synthesis of Compound **11**

Compound **7** (300 mg, 0.44 mmol), *tert-*butyl (3-aminopropyl)(2-((*tert-*butoxycarbonyl)amino)ethyl)carbamate (**8**, 698 mg, 2.2 mmol), Et_3_N (61 μL, 0.44 mmol) were refluxed in EtOH (12 mL). After 5 h (TLC monitoring: PE/AcOEt, 1:1), the system was cooled to r.t. and the solvents removed under reduced pressure. The crude was purified over a silica gel column eluted with PE/AcOEt, 1:1 [40]. The fractions containing the title compound were collected and evaporated to afford pure **11**. White foam (76% yield). ^1^H-NMR (400 MHz; CDCl_3_) δ 8.30 (s, 1H, 2-H), 8.14 (d, *J* = 8.0 Hz, 2H, arom.), 7.96 (t, *J* = 9.2 Hz, 4H, arom.), 7.56 (ddt, *J* = 30.4, 13.6, 7.3 Hz, 5H, arom.), 7.37 (q, *J* = 8.4 Hz, 4H, arom.), 7.05 (s, 1H, 8-H), 6.69 (complex signal, 2H, 1′-H and NH), 6.11–6.04 (complex signal, 2H, 2′-H and 3′-H), 5.12 (bs, 0.3H, NHBoc), 4.99 (bs, 0.7H, NHBoc), 4.84 (bd, *J* = 11.9 Hz, 1H, 5′-H_a_), 4.75 (bs, 1H, 4’-H), 4.67 (dd, *J* = 12.0, 3.2 Hz, 1H, 5’-H_b_), 3.59 (bd, *J* = 4.1 Hz, 2H, C*H*_2_NH), 3.42–3.20 (complex signal, 6H, 2 × C*H*_2_NBoc and C*H*_2_NHBoc), 1.96–1.78 (m, 2H, CH_2_), 1.57–1.35 (complex signal, 18H, 2 × Boc). ^13^C-NMR (101 MHz; CDCl_3_) δ 166.1, 165.4, 165.1, 156.3, 156.2, 156.0, 153.2, 149.5, 133.6, 133.4, 129.8, 129.8, 129.7, 129.3, 128.7, 128.5, 128.4, 119.6, 102.5, 89.2, 89.1, 85.5, 80.2, 79.3, 79.0, 73.9, 71.4, 63.8, 46.7, 45.8, 44.4, 39.5, 38.4, 37.4, 29.7, 28.9, 28.3. UV (MeOH) *λ*_max_ 275, sh. 230 nm. ESI-MS *m/z* 957 ([M + H]^+^, C_47_H_54_BrN_6_O_11_, requires 957).

#### 2.2.2. Synthesis of Compound **12**

To a solution of compound **11** (320 mg, 0.33 mmol) in 20 mL MeOH, NaOAc (13 mg, 0.33 mmol) and Pd/C (10% *w/w*, 106 mg, 0.1 mmol) were added in a Parr reactor from which the air was removed by insufflating/degassing H_2_. This apparatus was charged with H_2_ at a pressure of 10 atm and the reaction system was allowed to stand for 2 h at r.t. under stirring (TLC monitoring was achieved by PE/AcOEt, 1:1). Afterwards, H_2_ was removed and the mixture was filtered over a Celite 545 pad, subsequently washed with 3 × 10 mL MeOH. After solvent evaporation under reduced pressure, the obtained crude was diluted with 30 mL AcOEt and extracted with saturated brine (3 × 30 mL). The separated organic layer, dried on Na_2_SO_4_ and filtered, was concentrated by rotary evaporation furnishing a crude containing compound **12** used for the next reaction without purification. Oil (99%). ^1^H-NMR (400 MHz; CDCl_3_) δ 8.34 (s, 1H, 2H, 2-H), 8.14 (d, *J* = 7.6 Hz, 2H, arom.), 7.96 (dd, *J* = 14.6, 7.7 Hz, 4H, arom.), 7.61–7.32 (m, 9H, arom.), 7.09 (d, *J* = 3.5 Hz, 1H, 8-H), 6.74 (d, *J* = 6.0 Hz, 1H, 1′-H), 6.65–6.40 (complex signal, 2H, 7-H and NH), 6.19 (apparent t, *J* = 5.9 Hz, 1H, 2′-H), 6.11 (apparent t, *J* = 4.8 Hz, 1H, 3′-H), 5.66 (bs, 0.3 H, NHBoc), 5.18 (bs, 0.7H, NHBoc), 4.83 (dd, *J* = 12.0, 2.4 Hz, 1H, 5′-H_a_), 4.79–4.72 (m, 1H, 4′-H), 4.67 (dd, *J* = 11.9, 3.6 Hz, 1H, 5′-H_b_), 3.63–3.52 (m, 2H, C*H*_2_NH), 3.37–3.20 (complex signal, 6H, 2 × C*H*_2_NBoc and C*H*_2_NHBoc), 1.95–1.73 (m, 2H, CH_2_), 1.48–1.41 (complex signal, 18H, 2 × Boc). ^13^C-NMR (101 MHz; CDCl_3_) δ 166.2, 165.4, 165.1, 156.4, 156.3, 155.9, 152.4, 150.3, 133.6, 133.3, 129.8, 129.8, 129.7, 129.4, 128.7, 128.5, 128.4, 128.4, 120.5, 104.2, 100.3, 85.5, 80.4, 79.9, 79.4, 73.7, 71.6, 64.1, 46.6, 43.9, 39.4, 38.6, 30.3, 28.3. UV (MeOH) *λ*_max_ 274 nm, sh. 230 nm. ESI-MS *m/z* 879 ([M + H]^+^, C_47_H_55_N_6_O_11_, requires 879).

#### 2.2.3. Synthesis of Compound **13**

To a solution of compound **12** (291 mg, 0.33 mmol) in 3.0 mL MeOH, NaOMe (89 mg, 1.6 mmol) was added and the obtained system was stirred for 2 h at r.t. (TLC monitoring was performed by AcOEt/MeOH, 9:1). After solution neutralization with a few drops of glacial acetic acid, solvents were removed under reduced pressure. The resulting crude was diluted with 30 mL AcOEt and extracted with 3 × 30 mL saturated brine. The separated organic layer, dried on Na_2_SO_4_, was filtered and concentrated by rotary evaporation. Crude purification was performed on a silica gel column eluted with increasing amounts of MeOH in AcOEt (up to 10%). The fractions containing the title compound were collected and evaporated to afford pure **13**. Foam (80%). ^1^H-NMR (400 MHz; CDCl_3_) δ 8.05 (s, 1H, 2-H), 6.97 (d, *J* = 3.4 Hz, 1H, 8-H), 6.58 (bs, 1H, NH), 6.41 (s, 1H, 7-H), 5.70 (d, *J* = 7.3 Hz, 1H, 1′-H), 5.07 (dd, *J* = 7.3, 5.1 Hz, 1H, 2′-H), 4.91 (bs, 1H, NHBoc), 4.43 (apparent d, *J* = 5.1 Hz, 1H, 3′-H), 4.31–4.28 (m, 1H, 4′-H), 3.93 (dd, *J* = 12.7, 1.7 Hz, 1H, 5′-H_a_), 3.74 (dd, *J* = 12.6, 1.4 Hz, 1H, 5′-H_b_), 3.62–3.47 (m, 2H, C*H*_2_NH), 3.43–3.20 (complex signal, 6H, 2 × C*H*_2_NBoc and C*H*_2_NHBoc), 1.95–1.73 (m, 2H, CH_2_), 1.54–1.40 (complex signal, 18H, 2 × Boc). ^13^C-NMR (101 MHz; CDCl_3_) δ 156.5, 156.3, 155.9, 150.8, 147.5, 124.9, 105.6, 98.2, 92.9, 86.7, 80.6, 79.5, 73.0, 72.4, 63.3, 46.6, 44.1, 39.3, 29.7, 28.4. UV (MeOH) *λ*_max_ 276 nm, sh. 215 nm. ESI-MS *m/z* 567 ([M + H]^+^, C_26_H_43_N_6_O_8_, requires 596).

#### 2.2.4. Synthesis of Compound **14**

To a solution of compound **13** (50 mg, 0.088 mmol) in CH_2_Cl_2_ (0.5 mL), cooled at 0 °C, 0.5 mL TFA was added in one portion dropwise. The reaction system was warmed to r.t. and allowed to stand for 2 h under stirring (TLC monitoring was performed by CH_2_Cl_2_/MeOH, 8:2) [40]. After solvent removal under reduced pressure, the obtained crude was dissolved in water and applied on a glass column containing Dowex-OH^–^ resin (10 equiv.). After elution, first with H_2_O and then with MeOH, we collected the fractions containing compound **14** and removed solvents under reduced pressure. Oil (99%). ^1^H-NMR (400 MHz; CD_3_OD) δ 8.10 (s, 1H, 2-H), 7.25 (d, *J* = 3.7 Hz, 1H, 8-H), 6.56 (d, *J* = 3.6 Hz, 1H, 7-H), 5.95 (d, *J* = 6.6 Hz, 1H, 1′-H), 4.66 (dd, *J* = 6.4, 5.4 Hz, 1H, 2′-H), 4.26 (dd, *J* = 5.2, 2.6 Hz, 1H, 3′-H), 4.12–4.08 (m, 1H, 4′-H), 3.84 (dd, *J* = 12.4, 2.6 Hz, 1H, 5′-H_a_), 3.71 (dd, *J* = 12.4, 2.8 Hz, 1H, 5′-H_b_), 3.58 (t, *J* = 6.8 Hz, 2H, CH_2_NHC=N), 2.73 (t, *J* = 6.1 Hz, 2H, CH_2_NH_2_), 2.69–2.59 (complex signal, 4H, 2 × CH_2_NH ), 1.86 (quintet, *J* = 7.0 Hz, 2H, CH_2_). ^13^C-NMR (101 MHz; CD_3_OD) δ 158.1, 152.0, 149.7, 124.6, 105.9, 100.0, 91.6, 87.3, 75.3, 72.7, 63.7, 52.7, 47.9, 41.8, 39.6, 30.5. UV (MeOH) *λ*_max_ 276 nm. ESI-MS *m/z* 367 ([M + H]^+^, C_16_H_27_N_6_O_4_, requires 367).

#### 2.2.5. Synthesis of Complex **6**

Compound **14** (32 mg, 0.088 mmol) was dissolved in 1.5 mL of a H_2_O/MeOH (1:1, *v/v*) solution; K_2_PtCl_4_ (36 mg, 0.088 mmol) was added and the solution was stirred in the dark at r.t. for 16 h. The precipitate **6** was collected by filtration, washed sequentially with H_2_O, MeOH, Et_2_O and then dried. Amorphous pale yellow solid (60%). ^1^H-NMR (400 MHz; DMSO-*d*_6_) δ 8.18 (s, 1H, 2-H), 7.71 (bt, *J* = 5.4 Hz, 1H, NHC=N), 7.39 (d, *J* = 3.4 Hz, 1H, 8-H), 7.04 (bs, 1H, NH), 6.66 (d, *J* = 3.6 Hz, 1H, 7-H), 6.22 (bs, 2H, NH_2_), 6.03 (d, *J* = 6.3 Hz, 1H, 1′-H), 5.33 (bt, *J* = 4.8 Hz, 1H, 5′-OH), 5.29 (d, *J* = 6.5 Hz 1H, 2′-OH), 5.16 (d, *J* = 4.7 Hz, 1H, 3′–OH), 4.48–4.43 (m, 1H, 2′-H), 4.16–4.10 (m, 1H, 3′-H), 3.94–3.92 (m, 1H, 4′-H), 3.68–3.51 (complex signal, 4H, 5′-H_a,b_ and CH_2_C=N), 3.36–2.56 (complex signal, 6H, 2 × C*H*_2_NH and C*H*_2_NH_2_), 2.20–1.88 (m, 2H, CH_2_). ^13^C–NMR (101 MHz; DMSO-*d*_6_) δ 157.0, 152.1, 150.2, 123.1, 104.3, 100.1, 88.4, 86.0, 74.6, 71.6, 62.7, 54.7, 49.9, 46.1, 38.4, 27.9. IR (KBr pellet) 3400, 3233, 2927, 1610, 1563, 1463, 1367, 1303, 1250, 1094, 1048, 896, 818, 726, 609, 552, 420 cm^−1^. UV (H_2_O) *λ*_max_ 278 nm, sh. 227 nm. HRESI-MS *m/z* 633.1093 ([M + H]^+^, C_16_H_27_Cl_2_N_6_O_4_Pt, requires 633.1120).

#### 2.2.6. Preparation of ODNs **15**, **16**

The complementary ODN sequences **15** and **16** (Table 1) were obtained by solid phase synthesis using *β*-cyanoethyl phosphoramidite chemistry on a Expedite 8909 DNA synthesizer. For the syntheses, we used a CPG Universal Support (35 mg, 1.4 µmol) with a 1 µmol scale protocol, with the DMT-OFF option. Subsequently, the oligomers were detached from the resin and deprotected by treatment with concentrated aqueous ammonia (T = 55 °C, t = 12 h). The combined filtrates and washings were concentrated under reduced pressure. The crudes were dissolved in H_2_O, purified by HPLC and desalted. The identity and purity of the ODNs were confirmed by HPLC (data not shown) and ESI-MS analyses (see spectra). The ODN concentration was determined spectrophotometrically at *λ* = 260 nm and 90 °C, using the molar extinction coefficient ε = 128.4 for **15** and ε = 96.6 cm^−1^ mM^−1^ for **16**, as determined using the Sigma-Aldrich OligoEvaluator^TM^ web tool (www.oligoevaluator.com).

#### 2.2.7. Preparation of Duplex **d15/16** and Incubation with Cisplatin, Tubercidin, Complexes **5b** and **6** and Diamine **14**

Duplex **d15/16** was prepared by mixing ODNs **15** and **16** in a 1:1 ratio at 400 µM concentration in 10 mM phosphate buffer solution (PBS) containing 50 mM NaCl buffer at pH = 7.0. The solution was heated at 95 °C for 5 min, then slowly cooled to r.t over 12 h (annealing procedure) [41] and equilibrated at 4 °C for at least 4 h. The resulting solution was finally diluted to 2 μM with 10 mM PBS/50 mM NaCl buffer before **5b**, **6**, **14**, cisplatin or tubercidin addition at 1:2 and 1:10 DNA/compound ratio. Stock solutions of **5b**, **6**, **14**, cisplatin and tubercidin (2 mM) were freshly prepared by dissolving the solid compounds in a 20% DMSO containing 0.9% NaCl (154 mM NaCl) solution. After the dissolution, 5 μL of the 2 mM and 37.5 μL of the 1.3 mM of these samples were taken and rapidly left in contact with the **d15/16** solution, thus being rapidly diluted to the DMSO concentration of 0.04% and 0.3% in volume (to be used for the experiments involving 2 and 10 equiv., respectively). The solutions were incubated in the dark at 37 °C for 24, 48 and 72 h before data acquisition.

### 2.3. CD Spectroscopy

#### 2.3.1. CD Data Acquisition

CD spectra were acquired on a Jasco 1500 spectropolarimeter equipped with a Jasco PTC-348-WI temperature controller. All spectra were recorded at 37 °C in the 220–320 nm range, with a 1 cm path-length quartz cuvette at the 2 µM concentration, and averaged over 5 scans, with the following parameters: 1 s response time, 100 nm min^−1^ scan rate and 2 nm bandwidth. For each spectrum, the baseline due to the buffer was subtracted and the resulting spectra were normalized in order to have zero ellipticity at the 360 nm wavelength [42].

#### 2.3.2. CD Melting

Thermal denaturation experiments were performed by monitoring the CD values at 278 nm for **d14/15** in the 20–85 °C temperature range, with a temperature scan rate of 1 °C/min. The melting temperatures (T_1/2_) values have been obtained from the raw data by smoothing with an adaptive filter prior to calculate of the second derivatives using the software Jasco Spectra Manager^TM^ Suite.

### 2.4. Studies of Stability of the Complex **6**

A 0.5 mM solution of the complex **6** was prepared in a 0.9% NaCl solution containing a DMSO concentration of 0.5% (*v/v*) and incubated for 72 h at r.t. in the dark. The extent of degradation of the complex **6** was monitored through HPLC (see *General Methods*) after 24, 28 and 72 h, setting the UV detector at λ = 260 nm.

### 2.5. Biology

#### 2.5.1. Cell viability assay

Cells were plated in 96-well microplates (Corning) at the following densities: 2000 cells/well for WM266 and HDF, 1200 cells/well for HeLa, and 1000 cells/well for A375. After 24 h of incubation, cells were treated by increasing concentrations of synthetized compounds previously solubilized. In detail, the complex **6** and cisplatin were dissolved in 10% DMSO/0.9% NaCl, at 2 mM concentration, and then diluted immediately to the final 0.5% DMSO concentration with the cell growth medium whereas the diamine **14** and tubercidin were dissolved in H_2_O at 10 mM concentration. Cell viability was examined by using the 3-(4,5-dimethylthiazol-2-yl)-2,5-diphenyltetrazolium bromide (MTT, Sigma Aldrich) assay after 72 h of treatment. Subsequently, plates were analyzed by a microplate reader (Enspire, Perkin Elmer, USA) at 570 nm. The results, given as percentages of viable cells in respect to the control (vehicle treated cells), are expressed as means (±SE) of, at least, three independent experiments (each performed in triplicate). The statistical significance of the differences, evaluated for the anti-proliferative assay, was analyzed by Student’s *t* test, paired, two-sided; a *p* < 0.05 was considered significant. The IC_50_ value of each compound was calculated by GraphPad Prism software.

#### 2.5.2. Apoptosis Assay

The apoptosis analysis was performed on HeLa cells seeded at 4 × 10^4^ cells/mL in a 6-well plate. The cells were treated in the absence or presence of IC_50_ concentration of examined compounds at 37 °C and apoptosis was analyzed after 72 h by double staining with annexin V/FITC and propidium iodide (PI) (eBioscience, USA) [43]. The cells undergoing apoptosis were quantified using a flow cytometer equipped with a 488 nm argon laser (Becton Dickinson, USA) by Cell Quest software. All FACS analyses were performed at least 2 times.

## 3. Results and Discussion

### 3.1. Chemistry

**Scheme 1:**
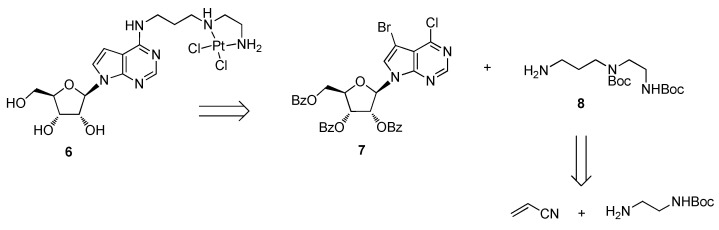
Retrosynthetic analysis for the preparation of the Pt(II) complex **6**.

**Scheme 2:**
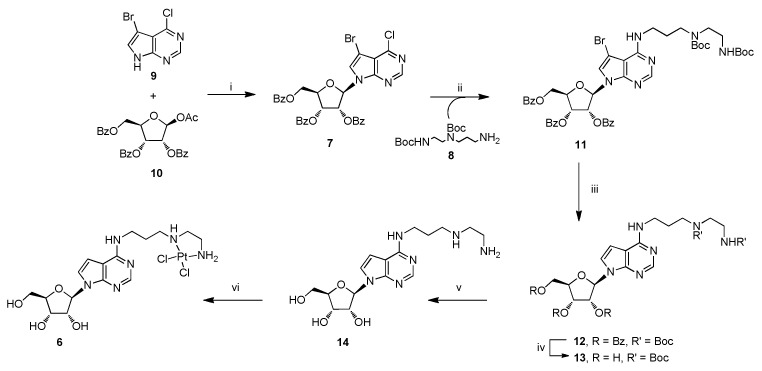
Reagents and conditions: (i) [44]; (ii) **8**, EtOH, 5 h, reflux; (iii) MeOH, NaOAc, H_2_/Pd/C, 2 h, r.t.; (iv) NaOMe, MeOH, 2 h, r.t; (v) (a) Trifluoroacetic acid (TFA)/CH_2_Cl_2_, 1:1 (*v/v*), 2 h, r.t.; (b) Dowex-OH^−^ resin, H_2_O/MeOH; (vi) K_2_PtCl_4_, H_2_O/MeOH 1:1, 16 h, r.t., in the dark.

The retrosynthetic analysis (Scheme 1) shows that the platinum complex **6** can be obtained by employing as starting materials the protected 7-bromo-6-chloro-7-deazapurine-β-d-riboside sugar (**7**) and *tert-*butyl (3-aminopropyl)(2-((*tert-*butoxycarbonyl)amino)ethyl)carbamate (**8**). The former can be obtained stereoselectively by following the procedure of Seela F. et al. through the coupling of 7-bromo-6-chloro-7-deazapurine (**9**, Scheme 2) with 1-*O*-acetyl-2,3,5-tri-*O*-benzoyl-*β*-d-ribofuranose (**10**) [44], whereas the compound **8** can be readily prepared in three steps in very high yields by following the procedure of Eisenfu, A. et al. [45] that starts from Michael addition of *tert*-butyl (2-aminoethyl)carbamate to acrylonitrile, followed by Boc protection of the secondary amino group and reduction of the nitrile to a primary amine.

Compound **8** was then reacted with the nucleoside **7** in ethanolic solution under reflux; differently from our previous reports on similar reactions [34,35], compound **11** did not precipitate after cooling, and the product was isolated only after column chromatography (76% yield). Next, the C7-Br bond in **11** was hydrogenated in the presence of H_2_/Pd/C and the crude was directly subjected to the sugar deprotection (Zemplen conditions, NaOMe/MeOH), yielding compound **13** (60% over two steps). The removal of Boc protecting groups on **13** was performed with trifluoroacetic acid (TFA) [46] and the crude was then passed over a Dowex-OH^–^ resin to quantitatively recover the free diamine **14**.

The latter was then reacted with K_2_PtCl_4_, affording the platinum complex **6** as a pale-yellow solid that was isolated by filtration in a 60% yield. The structures of all intermediates and of platinum complex **6** were determined by NMR and ESI-MS analyses. In detail, the platination of the diamino ethane moiety in the complex **6** was confirmed by the downfield shift of the ^13^C resonances of CH_2_NH_2_ (Δδ ≈ 5 ppm) and CH_2_NH (Δδ ≈ 3 ppm each) compared with the same signals displayed by the diamine **14**. Conversely, the platination of C6NH, N1 and N3 nitrogen atoms could be excluded, as no significative differences between the chemical shift of carbons belonging to the purine rings of the two compounds were observed [47]. In the HRESI-MS(+) spectrum of complex **6**, the base peak corresponded to the protonated bis-chlorinated species; in addition, traces of the fragment ion [M − Cl]^+^ (*m/z* = 596.1347) were also detected.

### 3.2. Study of the Interaction of the Platinum Complex **6** with the Model Duplex DNA **d15/16** through CD Spectroscopy

It is widely accepted that the target of cisplatin is the nuclear DNA and the apoptotic effects are induced by the formation of stable covalent adducts with adjacent guanines located essentially on the same strand [4]. Accordingly, we monitored, through CD spectroscopy, the interaction of the complex **6**, the diamine **14**, tubercidin and cisplatin with a model duplex DNA having three “GG” boxes (**d15/16**, Table 1).

**Figure 3:**
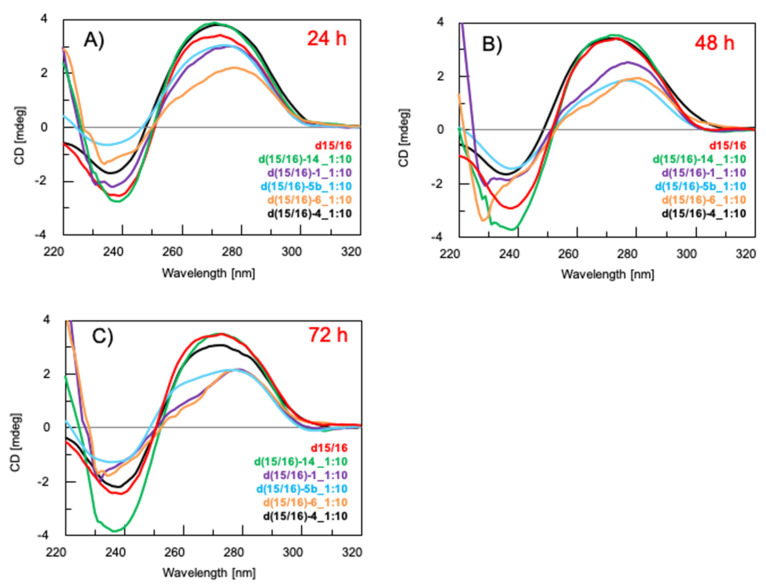
Overlapped CD spectra of **d15/16** recorded at 2 μM concentration (red line) before and after 24 (panel **A**), 48 (panel **B**) and 72 h (panel **C**) of incubation with 10 equiv. of **14** (green line), cisplatin **1** (violet line), **5b** (light blue), **6** (orange line) and tubercidin **4** (black line) at 37 °C. All samples were dissolved in 10 mM PBS/50 mM NaCl-containing buffer at pH 7.0 and the spectra were normalized at 320 nm.

In addition, we also monitored the DNA reactivity of the complex **5b** (n = 2), which shares the alkyl chain length separating the nucleoside scaffold from the cisplatin-like unit with the complex **6**. The DNA duplex **d15/16**, reported in the literature for the study of novel Pt(II) complexes [48,49], was obtained by hybridizing equimolar amounts of oligodeoxynucleotides (ODNs) **15** and **16**. In parallel experiments, the complexes **5b** and **6**, the diamine **13**, tubercidin and cisplatin were incubated in two and ten equiv. excess [48,49] with respect to the duplex **d15/16** and the CD spectra of the resulting mixtures were acquired after 24, 48 and 72 h of incubation. The CD spectrum of the **d15/16** dissolved in 10 mM PBS/50 mM NaCl (pH = 7.0) showed a negative band at 239 nm due to the characteristic helicity of the right-handed B form and a positive band at 273 nm, indicative of base-stacking (Figure 3) [22,50]. In the presence of two equiv. of **5b, 6, 14**, tubercidin and cisplatin, no significant differences in the CD spectrum of **d15/16** were found at all time points (Figure S17, Supplementary Material). Conversely, by increasing the ratio from two to ten equiv., relevant conformational changes were observed after incubation of **d15/16** with the platinated complexes **5b** and **6** and cisplatin (Figure 3). In detail, all complexes induced a disruption of the B-DNA double helix, as evidenced by a reduction of the 273 nm band together with hypsochromic and bathochromic shifts of the negative and positive signals, respectively (Tables S1–S3, Supplementary Material).

These destabilizing effects are indicative of non-intercalative interactions of the complexes with the double-stranded DNA [50]. The most pronounced changes were elicited by complex **6** after 24 and 48 h of incubation (Tables S1–S2). In fact, we observed a reduction in maximum intensity of 36% at 24 h and 49% at 48 h, compared to the untreated duplex. In the case of cisplatin, the reduction was 8% and 26%, whereas for the complex **5b,** the reduction was 6% and 44%, respectively. Interestingly, the CD profiles obtained after 72 h of incubation of complex **6** and cisplatin with **d15/16** matched, leaving us to speculate that both the complexes could have a similar mechanism of action. The above reported CD data unveil the different binding modes of **6** and cisplatin for the studied DNA duplex sequence. We believe that the greater steric hindrance of **6** favors its interaction with the terminal “GG“ boxes of the target duplex model at earlier time points, which in turn induces the partial unzip of the double helix as resulting from the observed reduction in the molar ellipticity of the dichroic signals at 239 and 273 nm. Conversely, at 72 h from the incubation, we did not observe significant variation in the CD profiles of the two complexes, thus indicating that the binding saturation of the “GG“ boxes for both cisplatin and **6** occurred. To collect further information on the stoichiometry and stability of the complexes formed by **6** and cisplatin with the **d15/16** at 24 and 48 h, we performed CD melting experiments. As expected, the melting curves (Figure S18) evidenced the loss of the duplex structure, with a gradual reduction in the dichroic signal not accompanied by any appreciable inflection point at all the studied time points. Taking into account the differences in the shape of the CD melting profiles recorded at 24 and 48 h, we hypothesize that **6** is faster than cisplatin in reacting with the three “GG“ boxes of the **d15/16**. In particular, its binding with the terminal “GG“ boxes could be responsible for the lower mdeg values at 239 and 273 nm observed in the CD profiles recorded at 24 and 48 h [22,50]. Regarding the effects of the incubation of the diamine **14** and tubercidin with **d15/16**, only weak increases in the intensity of the positive band at 273 nm were detected after 24 h; conversely, a more relevant increase in the intensity of the negative band at 245 nm was observed in the case of the interaction of the duplex with the compound **14** (Figure 3) at all time points. The latter result is likely a consequence of the greater number of polar interactions that diamine **14** is able to form with the DNA double helix [51,52]. Lastly, both the diamine **14** and tubercidin did not appreciably affect the melting profile of **d15/16** over the 72 h incubation period (Figure S19). This study of interaction of tubercidin with a model DNA duplex through CD spectroscopy is unprecedented and allows us to assert that the DNA is not its biological target.

### 3.3. Study of Stability of the Complex **6** Dissolved in Physiological Solution

**Scheme 3:**
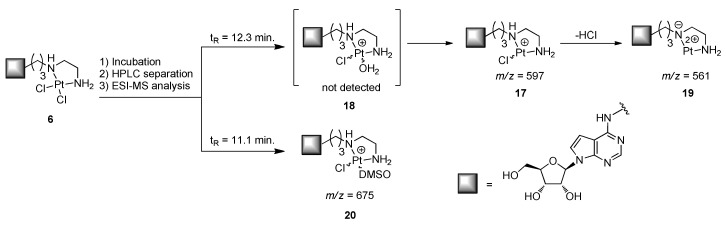
The proposed structures of the main species obtained after incubation of the complex **6** (0.5 mM) in a 0.9% NaCl solution containing 0.5% DMSO at r.t for 72 h.

The strong biological activity of cisplatin is attributable to the presence of the two reactive chloro ligands around the Pt atom, that can be replaced by several nucleophiles (water, sulfur-donors) in a biological medium. Studies of the stability of cisplatin, carboplatin and oxaliplatin in physiological conditions were recently reported by Varbanov et al. by using the high performance liquid chromatography–mass spectrometry (HPLC-MS) technique [53] and demonstrated that the nature of ligands around the Pt atom could deeply influence the reactivity of the drugs. As the novel complex **6** is a cisplatin mimic, we evaluated its stability in physiological conditions before testing the cytotoxic properties on tumor cell lines. To this aim, we incubated the complex **6** (0.5 mM) in a physiological solution (0.9% NaCl) with DMSO concentration of 0.5% for 72 h at r.t., and monitored its stability at 24, 48, 72 h through HPLC. The main species eluted from the column were collected, diluted with methanol, and analyzed by ESI-MS in positive mode. After 24 h incubation, the complex **6** mainly decomposed into two species, with retention times (t_R_) of 11.1 and 12.3 min. respectively (Figure S20). In the ESI-MS spectrum of the firstly eluted species we detected the ions at *m/z* 597 and 561, both possessing the characteristic Pt isotopic patterns of mono-positive ions. The structure of the ion at *m/z* 597 was assigned as **17** (Scheme 3), that could derive from the not detected mono-H_2_O complex (**18**), after a removal of the H_2_O molecule during the ionization stage in the mass spectrometer; whereas the ion at 561 (**19**) was a fragment, generated by the loss of HCl. In the ESI-MS spectrum of the species eluted later, we detected an ion at *m/z* 675, showing the characteristic Pt isotopic pattern of a mono-positive ion. The structure was consistent with that of the species **20**, derived from the complex **6** after displacement of a Cl^–^ by DMSO [54]. After 48 and 72 h incubations, the HPLC profiles remained almost unchanged, except for the formation of a species eluted at t_R_ = 9.7 min, whose structure has not been identified. The HPLC experiments carried out demonstrated that: (1) a complete consumption of the complex **6** took place after 24 h incubation, with the parallel appearance of several compounds, whose amounts changed over the 72 h incubation period; (2) DMSO does interact with the complex **6** even in a very dilute environment, according to a previous report [55].

### 3.4. Biology

**Figure 4:**
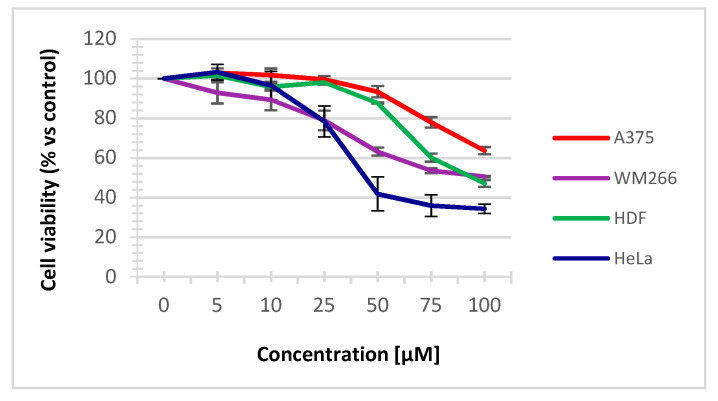
Cell viability assay of complex **6** on HeLa, A375, WM266, and human dermal fibroblasts (HDF) cell lines. The cells were incubated in the presence of compound at the indicated concentrations at 37 °C for 72 h. Cell viability was measured by using the MTT assay. The results are presented as the percentage of viable cells with respect to the control (vehicle treated cells) and are expressed as means ±SE of at least three independent experiments. A *p* ≤ 0.01 was observed from 50 to 100 μM for WM266 and HDF, at 75 and 100 μM for A375 and HeLa; a *p* < 0.05 was observed at 25 and 50 μM for HeLa.

To evaluate the cellular effect upon interaction with the model DNA double helix, biological experiments were carried out. Primarily, cytotoxicity profiles of the platinum complex **6** and its diamine precursor **14** were evaluated on three different human tumor cell lines, cervical adenocarcinoma cells (HeLa), low metastatic (A375) and metastatic melanoma cells (WM266), as well as on human dermal fibroblasts (HDF), using the MTT cell viability assay. HDF cells were used as a healthy cellular model to determine the selectivity of action of the examined compounds. For these experiments, cisplatin and tubercidin were used as the positive controls (Table 2 and Figure S21). In detail, cells were incubated for 72 h in the presence of complexes at increasing concentrations ranging from 5 to 100 µM for **6** and **14**, and from 0.3 to 20 µM for cisplatin and from 0.075 to 20 µM for tubercidin. Complex **6** and cisplatin were dissolved in 100% DMSO and immediately diluted with 0.9% NaCl aqueous solution to obtain the 2 mM stock solutions, which were diluted with the growth medium at the final concentrations containing less than 0.5% DMSO [34,35,50]. The small amount of DMSO, necessary to completely solubilize the Pt(II) complex, should not affect its biological activity, in accordance with the recent findings on the combination studies of cisplatin and DMSO on KB-3-1 or DLD-1 tumor cells lines [55]. The resulting dose–response curves, reported in Figure 4, show for complex **6** a concentration-dependent inhibition in all tested cell lines. 

In particular, the complex **6** presented a good effect on HeLa cell viability, which decreased to 35% at the highest tested concentration. Furthermore, the complex **6** proved less active on WM266 and only slightly effective on A375 and on healthy HDF, displaying an interesting tumor cell selectivity, the main feature for the development of therapeutic compounds. This selectivity of action was also evident in the evaluation of the IC_50_ values, as reported in Table 2, that indicated, for the examined complex, an IC_50_ in the medium micromolar range (55.1 μM) on HeLa, a lower effect towards WM266 (IC_50_ = 91.1 μM) and a low interference in the proliferation of A375 and HDF, showing both IC_50_ > 100 μM. The complex **6** proved less active than both cisplatin and tubercidin on all treated cells; however, equally, cisplatin and tubercidin did not display selectivity of action, also with respect to non-tumorigenic cells. Lastly, the diamine precursor **14** was completely inactive on all the screened cell lines (Table 2 and Figure S21).

**Figure 5:**
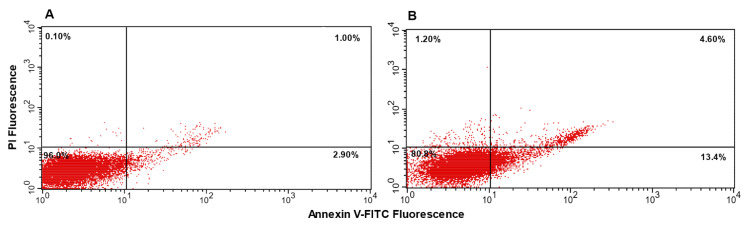
Apoptosis analysis with annexin V-FITC and propidium iodine (PI) double staining on HeLa cells. HeLa cells were seeded at 40,000 cells/well on a 6-well plate and treated with 50 µM of complex **6** for 72 h. Panel **A**: control (vehicle treated cells); Panel **B**: cells treated with the complex **6**. Lower left quadrant: viable cells; upper left: necrotic cells; upper right: advanced apoptotic cells; low right: early apoptotic cells. These pictures are representative of two independent experiments.

Apoptosis is a form of programmed cell death, where dead cells are split up into small pieces and are recycled by other cells. In contrast, death through necrosis induces the release of intracellular content and causes inflammation. Therefore, the involvement of the apoptosis process is a desirable feature in the therapeutic action. To evaluate whether the complex **6** was able to induce an apoptosis process, the annexin V/propidium iodine (PI) double staining assay was performed by flow cytometry analysis. Annexin V is an intracellular protein that binds to phosphatidylserine, that, during early apoptosis, trans-locates to the external leaflet of the cell membrane. FITC-labelled Annexin V can then be used to specifically identify apoptotic cells. However, Annexin V binding alone cannot differentiate between apoptotic cells and necrotic. Therefore, PI, at low concentrations, positively stains late-stage apoptotic cells and necrotic cells [56]. So, the necrotic cells will stain with only PI, advanced apoptotic cells with PI and FITC, and early apoptotic cells with only FITC. The assay was performed on HeLa cells, which proved to be the most sensitive cells in the proliferation assay. To this aim, cells were incubated with the complex **6** at 50 µM concentration. The results of the experiment show that 13% of cells treated with this complex were in early apoptosis (Figure 5, panel A) and 5% in advanced apoptosis (Figure 5, panel B), demonstrating that **6** was able to induce an apoptotic process, according to literature data concerning most of the platinated complexes [57], although to a lesser extent than cisplatin or tubercidin treatments (Figure S22).

## 4. Conclusions

The conjugation of cisplatin-like units to composite molecular scaffolds produced complexes endowed with interesting cytotoxic properties; accordingly, we focused our attention on the syntheses of Pt(II) complexes carrying modified nucleosides. With the aim of synthesizing neutral Pt(II) complexes mimicking the cisplatin reactivity, the 7-deazapurine riboside (tubercidin) was tethered at the C6 purine position through the metal-chelating diamino *N*-alkyl-amide and diamino *N*-alkyl linkers, thus obtaining the complexes **5** and **6**, respectively. Even though the complex **6** proved less cytotoxic than cisplatin against the tested cancer cell lines, the results of our CD investigations demonstrate that complex **6** reacted more quickly than cisplatin with the model duplex DNA **d15/16** at 24 and 48 h. Given the best propensity of complex **6** to induce conformational changes to the DNA double helix, we hypothesized that its weaker in vitro biological activity could be ascribed either to deactivation processes in intra/extracellular environments, before the Pt(II) complex can reach its specific target (DNA), or to a poor cellular uptake. Despite the lower cytotoxic activity of complex **6** relative to cisplatin, its greater selectivity toward tumor cells could be exploited in multi-drug therapy approaches. Furthermore, in the light of its selectivity of action and non-cytotoxicity on the normal HDF cell line, studies are underway to encapsulate the Pt(II) complex **6** in lipophilic macromolecular systems resistant to biological fluids. Lastly, the nucleoside-tethered diamine **14** could be used for the complexation of other metals useful in anticancer therapy, extending the role of nucleosides in the development of novel potential chemotherapeutics.

## Figures and Tables

**Table 1:** The oligodeoxynucleotides (ODNs) **15** and **16** used in this study to form the model duplex DNA **d15/16**.

ODN	Sequence
**15**	d(5’-GGAGACCAGAGG-3’)
**16**	d(5’-CCTCTGGTCTCC-3’)
**d15/16**	d(5’-GGAGACCAGAGG-3’) d(3’-CCTCTGGTCTCC-5’)

**Table 2:** IC_50_ values obtained after 72 h incubation with the examined compounds on HeLa, A375, WM266 and HDF cell lines.

Entry	IC_50_ [μM]			
**Cell lines**	**HeLa**	**A375**	**WM266**	**HDF**
**6**	55.1 ± 14.6	>100	91.0 ± 11.9	>100
**14**	>100	>100	>100	>100
**Cisplatin**	1.8 ± 0.75	1.3 ± 0.28	2.4 ± 0.40	6.3 ± 1.8
**Tubercidin**	0.11 ± 0.3	0.085 ± 0.023	0.076 ± 0.016	0.47 ± 0.2

## Supplementary Materials

The following are available online at https://www.mdpi.com/1999-4923/12/7/627/s1, Figures S1–S13: 1D- and 2D-NMR spectra of compounds **11**, **12**, **13, 14** and **6**, Figure S14: HR ESI-MS(+) spectrum of complex **6**, Figure S15: FT IR spectrum of complex **6**, Figure S16: ESI-MS(–) spectra of ODNs **15** and **16**, Figure S17: CD spectra, Tables S1–S3, Figures S18–S19: CD melting spectra, Figure S20: HPLC stability assay, Figure S21: Cell viability assays, Figure S22: Apoptosis assays.
