# Supplementary material for: Probing the DNA Reactivity and the Anticancer Properties of a Novel Tubercidin-Pt(II) Complex
Source: Pharmaceutics. 2020 Jul 4;12(7):627. doi: 10.3390/pharmaceutics12070627 (PMC7407906; doi:10.3390/pharmaceutics12070627)
Supplement: Supplementary file 1 [file pharmaceutics-12-00627-s001.pdf]

pharmaceutics

MDPI

Supplementary Material: Probing the DNA Reactivity and the Anticancer Properties of a Novel Tubercidin-Pt(II) Complex

Stefano D'Errico, Andrea Patrizia Falanga, Domenica Capasso, Sonia Di Gaetano, Maria Marzano, Monica Terracciano, Giovanni Nicola Roviello, Gennaro Piccialli, Giorgia Oliviero and Nicola Borbone

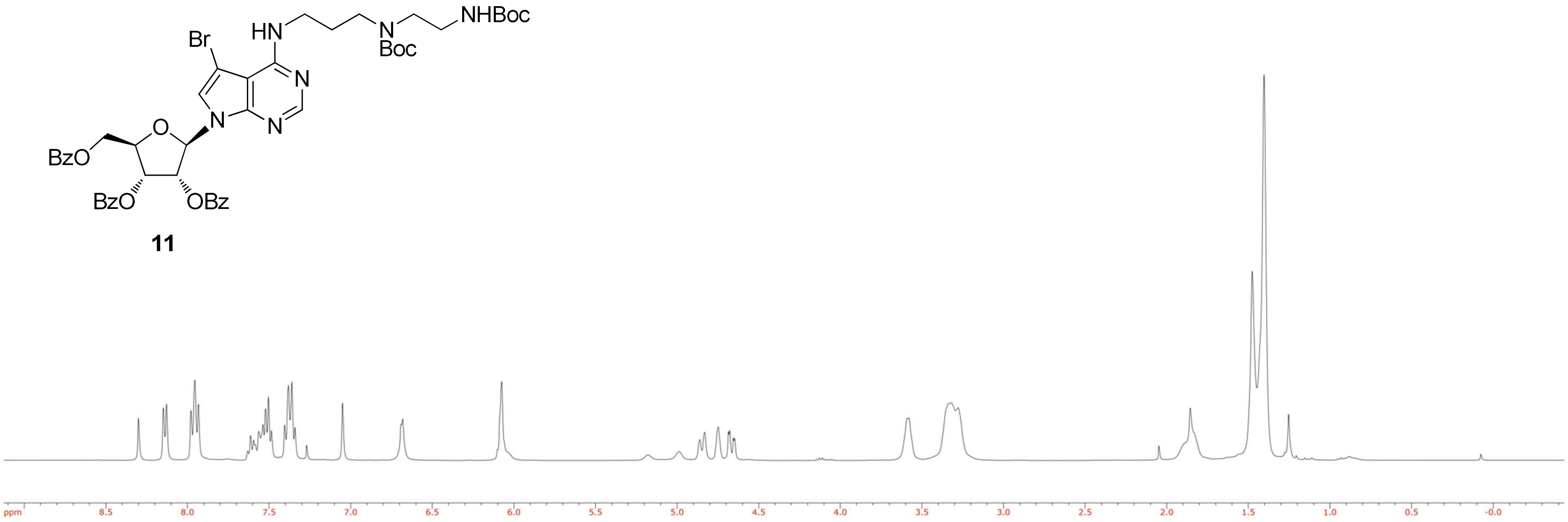

Figure S1. ^{1}H-NMR spectrum (400 MHz, $CDCl_3$) of compound **11.**

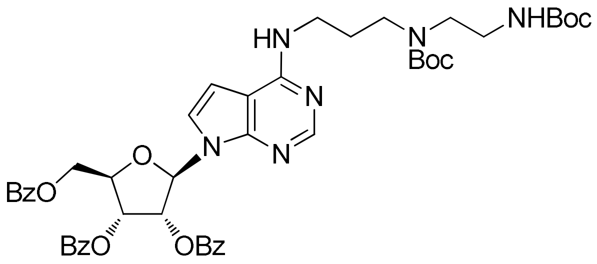

12

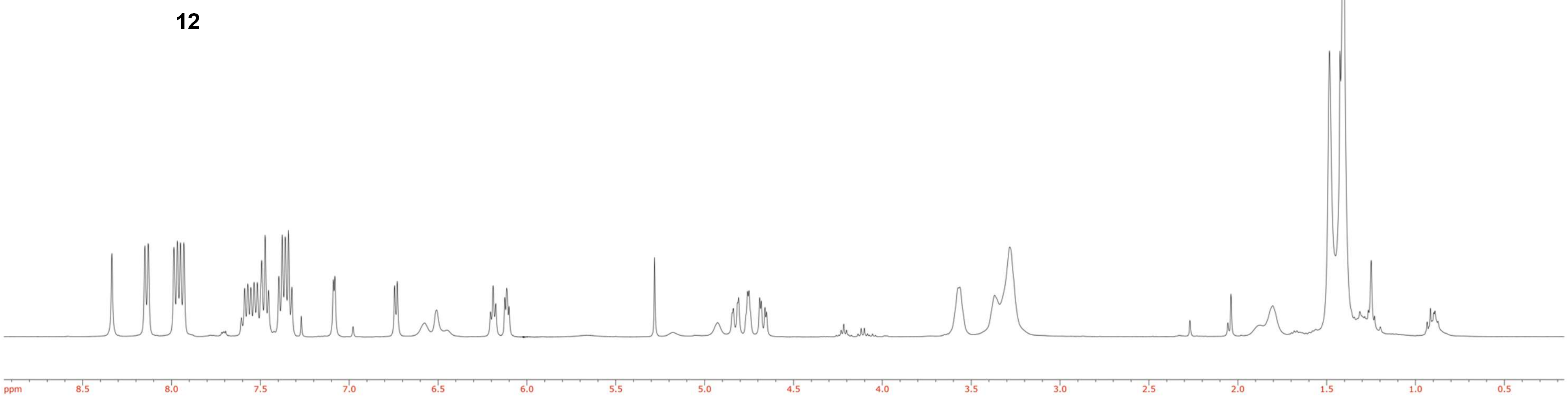

Figure S2. ^{1}H-NMR spectrum (400 MHz, $CDCl_3$) of compound **12.**

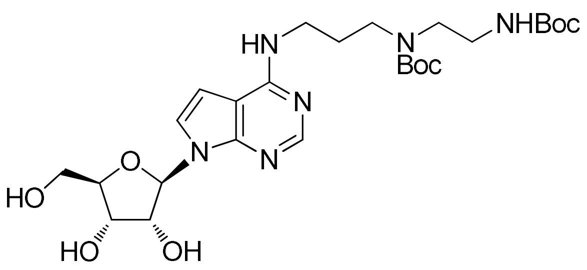

13

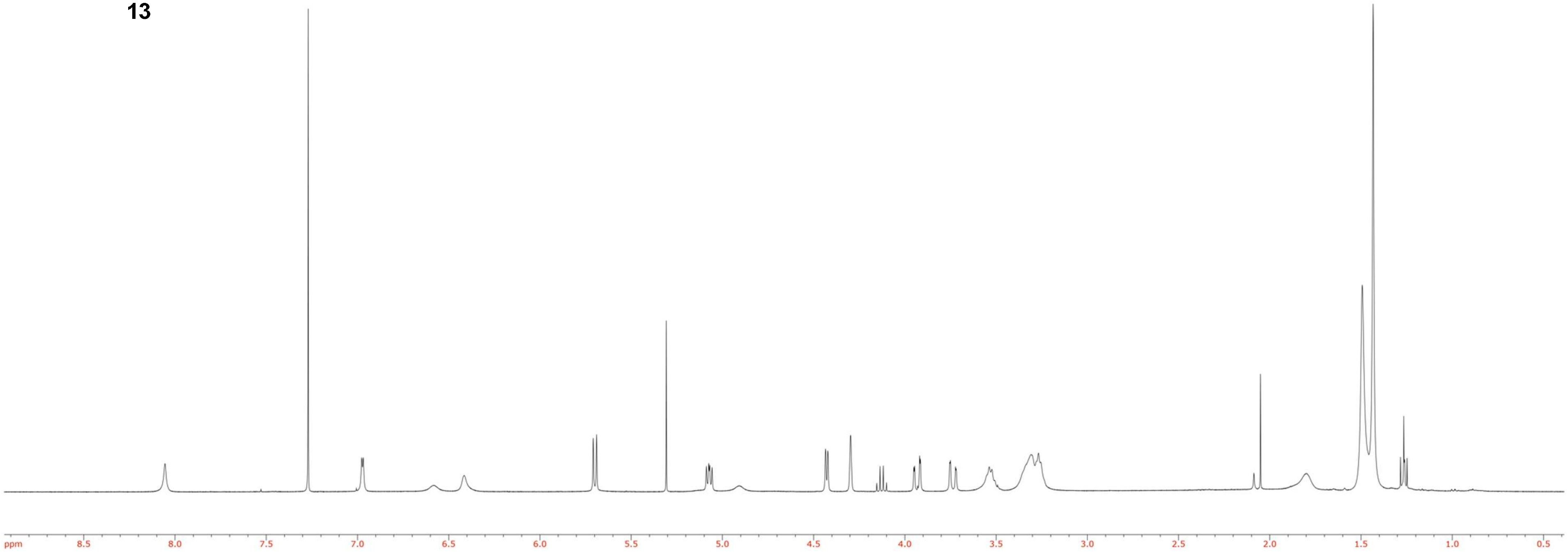

Figure S3. ^{1}H-NMR spectrum (400 MHz, $CDCl_3$) of compound **13.**

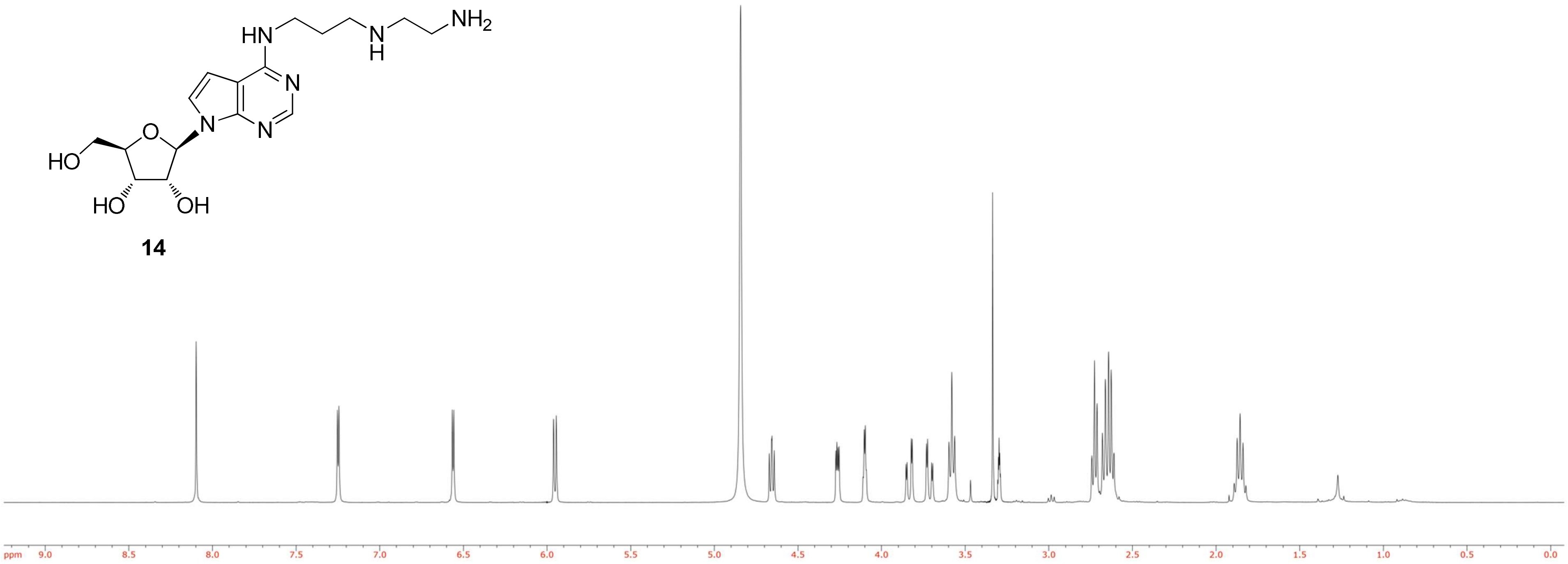

Figure S4. ^{1}H-NMR spectrum (400 MHz, CD_3OD) of compound **14.**

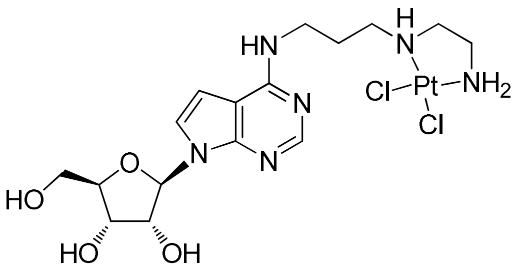

6

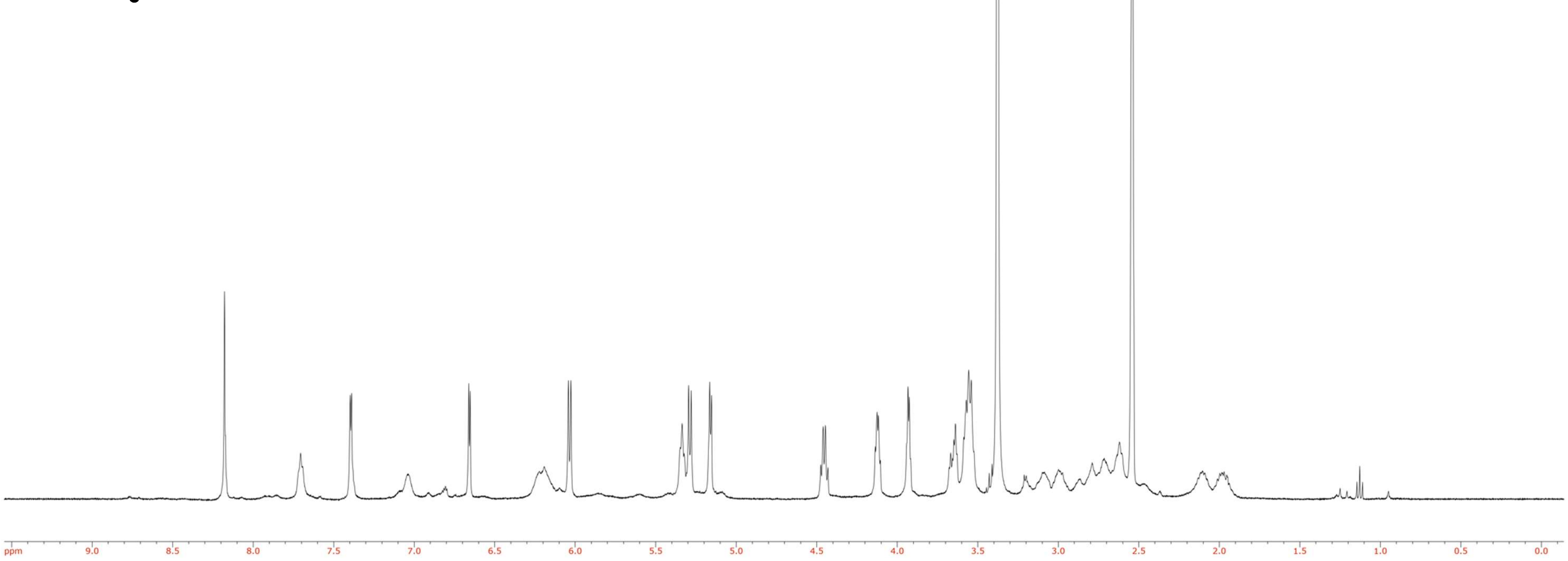

Figure S5. ^{1}H-NMR spectrum (400 MHz, DMSO-d_6) of complex **6.**

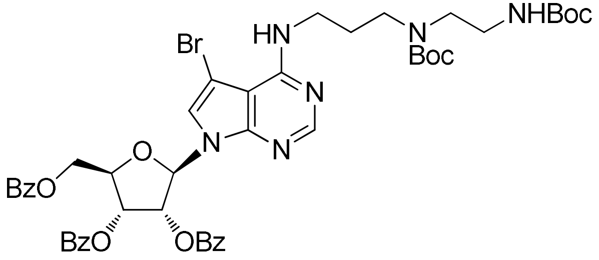

11

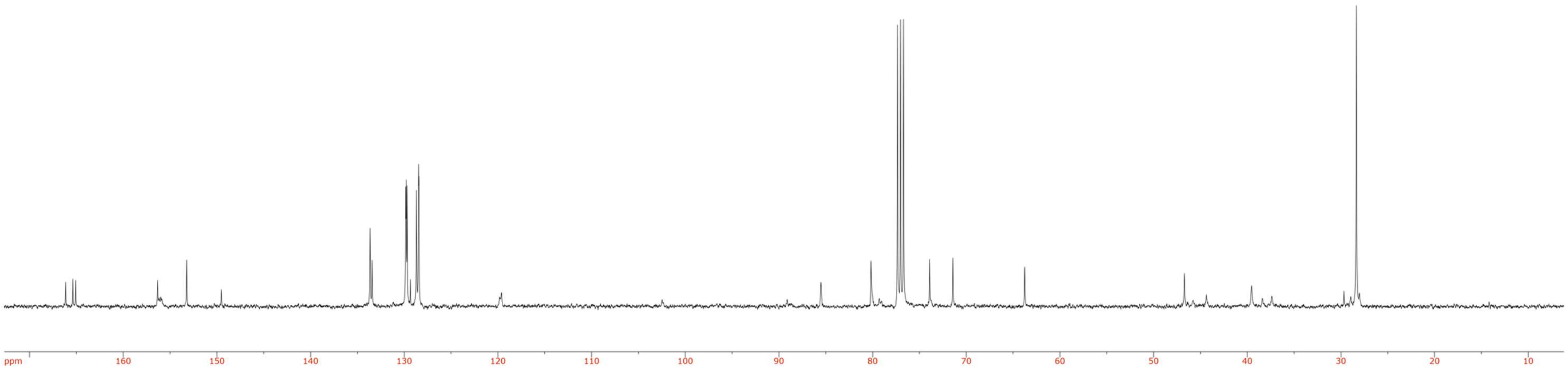

Figure S6. ^{13}C-NMR spectrum (100 MHz, $CDCl_3$) of compound **11.**

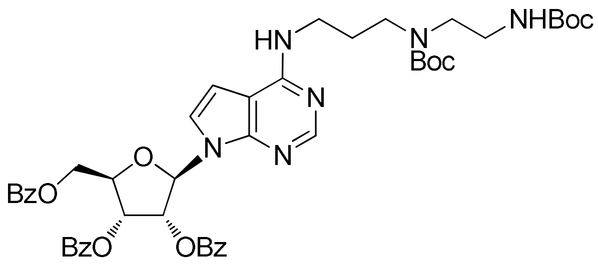

12

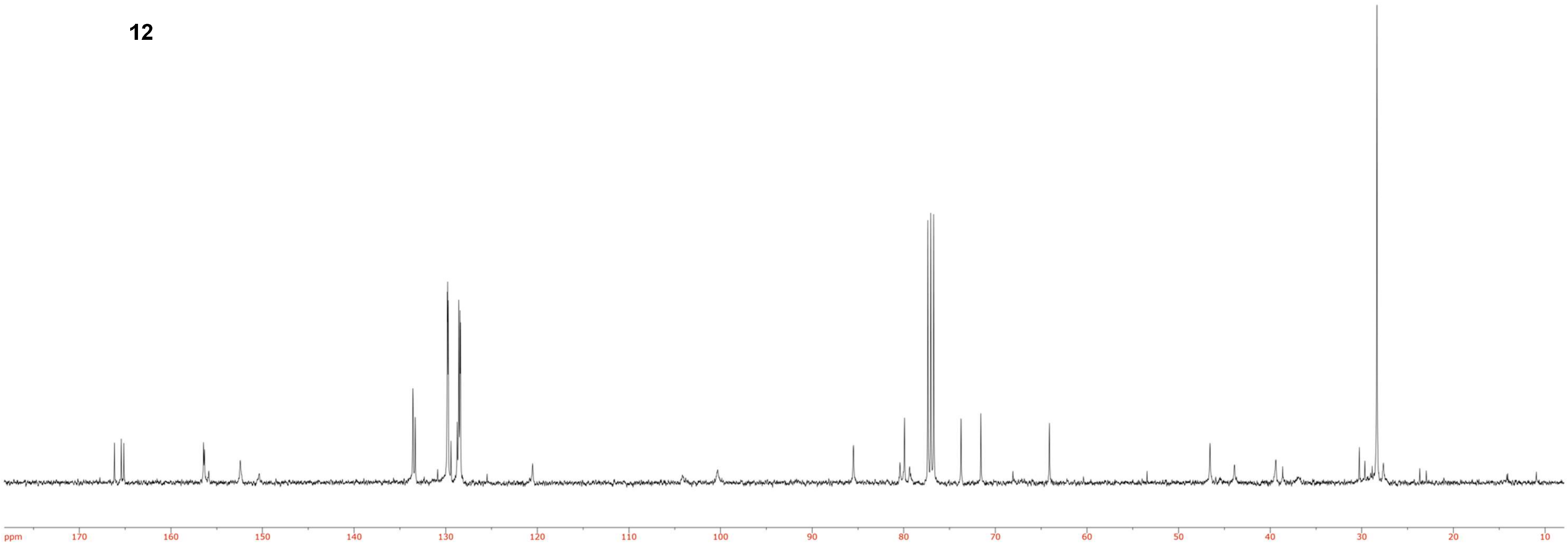

Figure S7. ^{13}C-NMR spectrum (100 MHz, $CDCl_3$) of compound **12.**

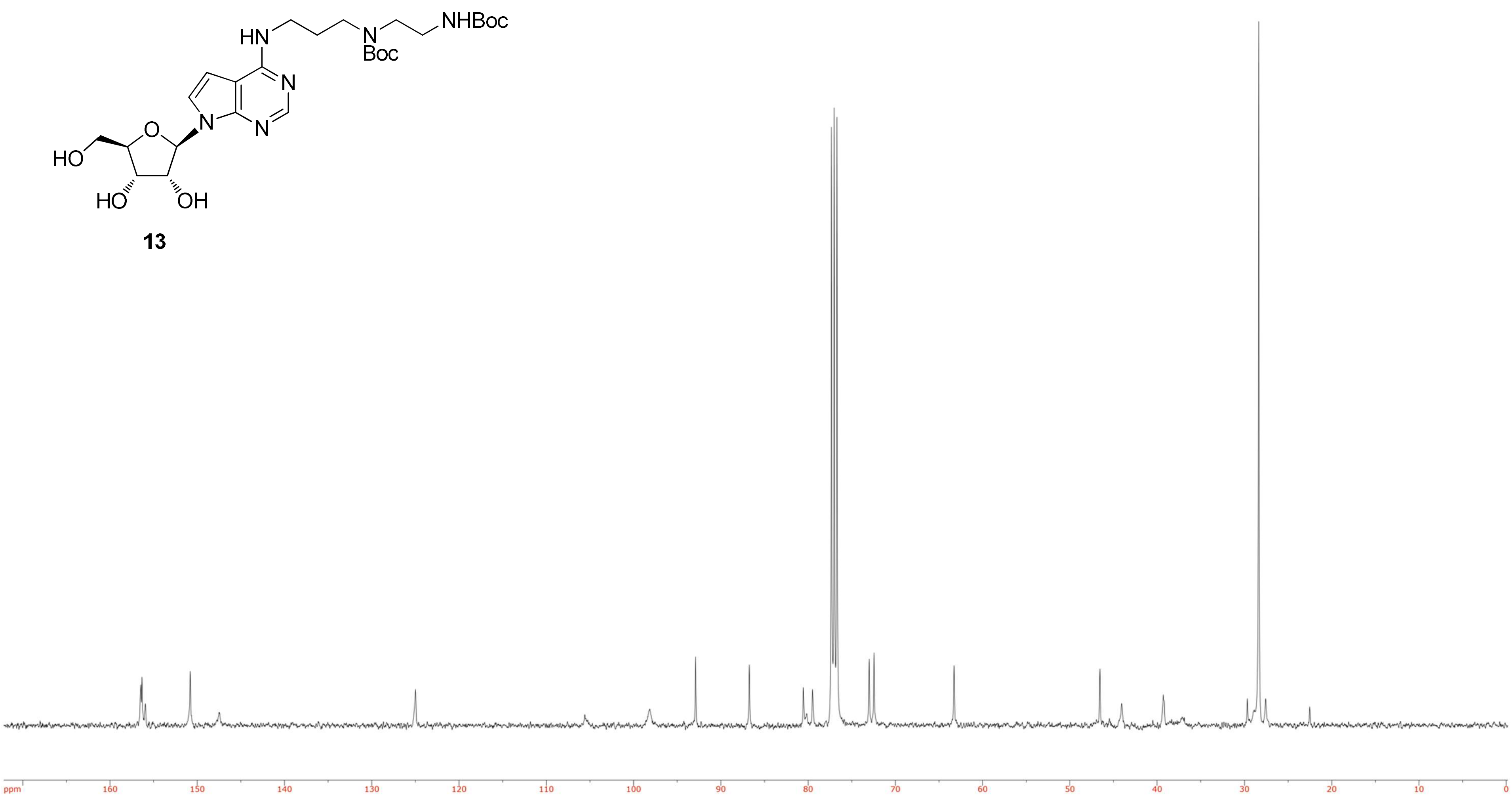

Figure S8. ^{13}C-NMR spectrum (100 MHz, $CDCl_3$) of compound **13.**

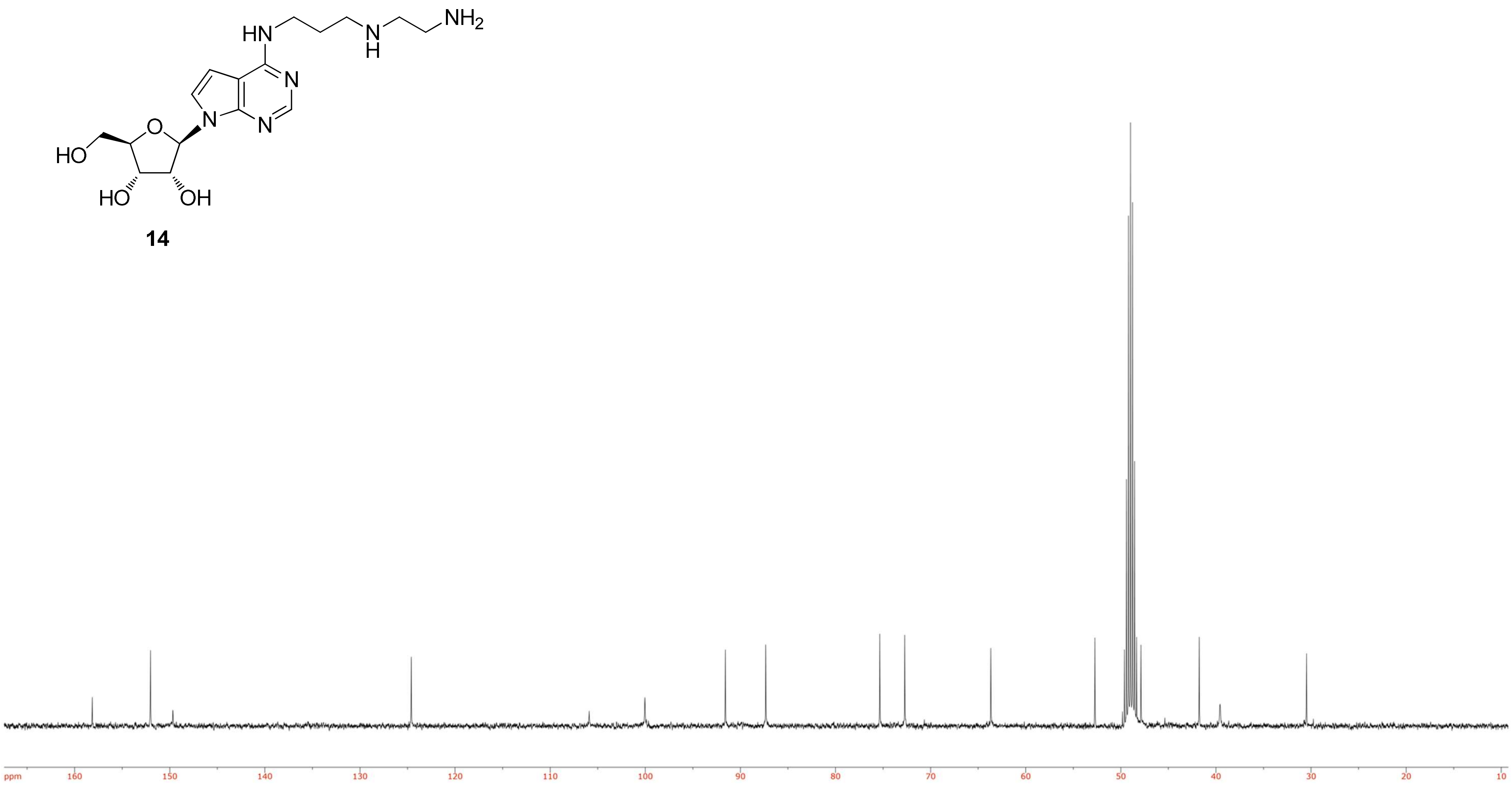

14

Figure S9. ^{13}C-NMR spectrum (100 MHz, CD_3OD) of compound **14.**

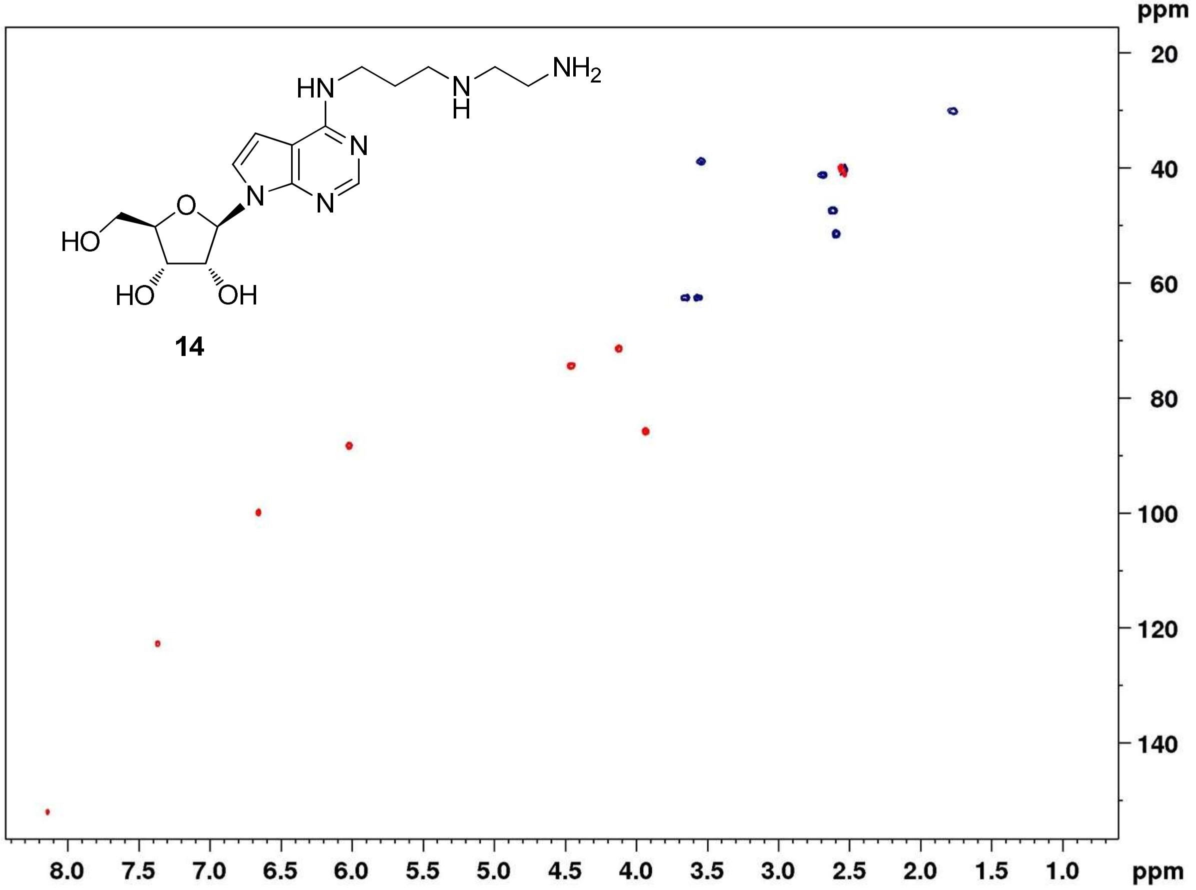

Figure S10. HSQC spectrum (100 MHz, CD_3OD) of compound **14.**

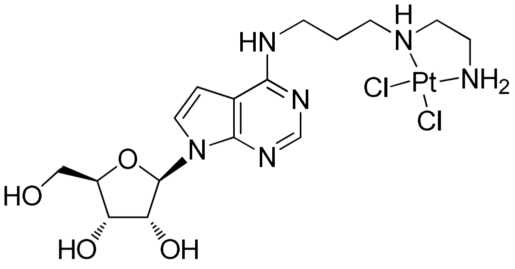

6

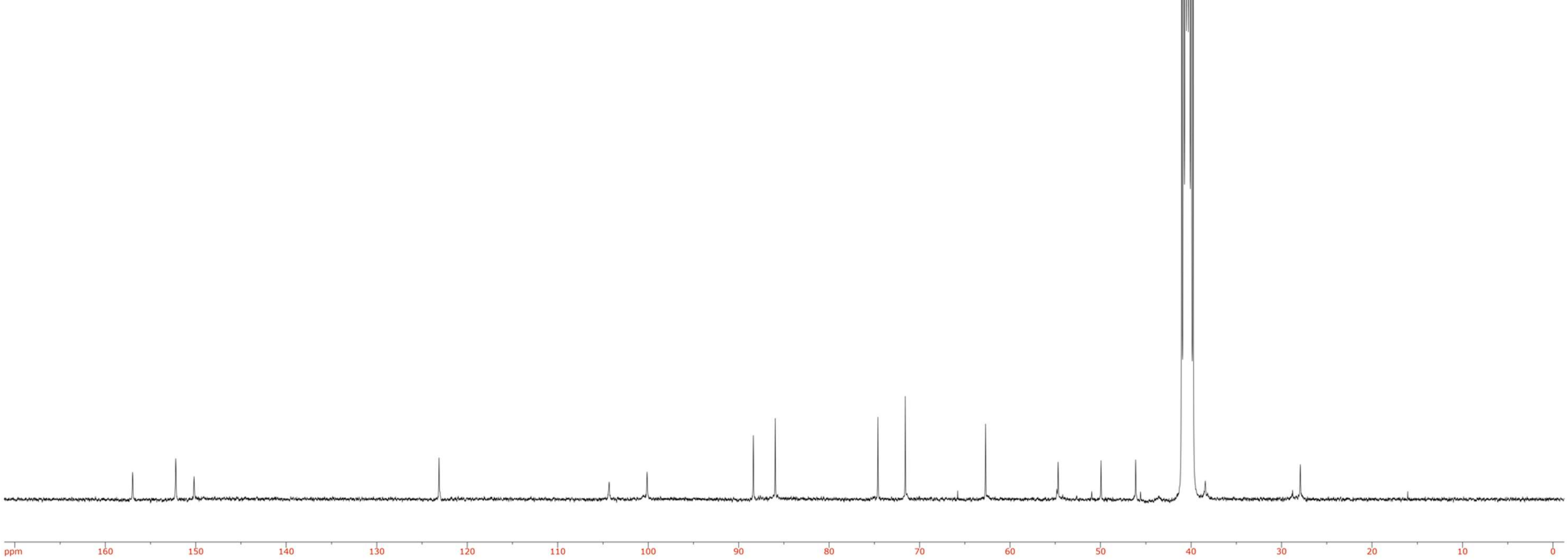

Figure S11. ^{13}C-NMR spectrum (100 MHz, DMSO-d_6) of complex **6.**

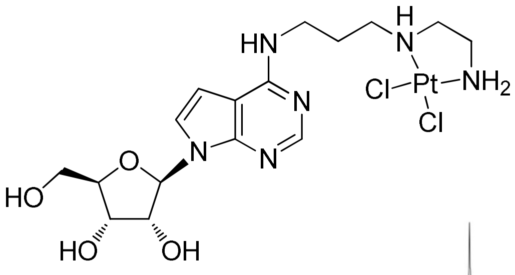

6

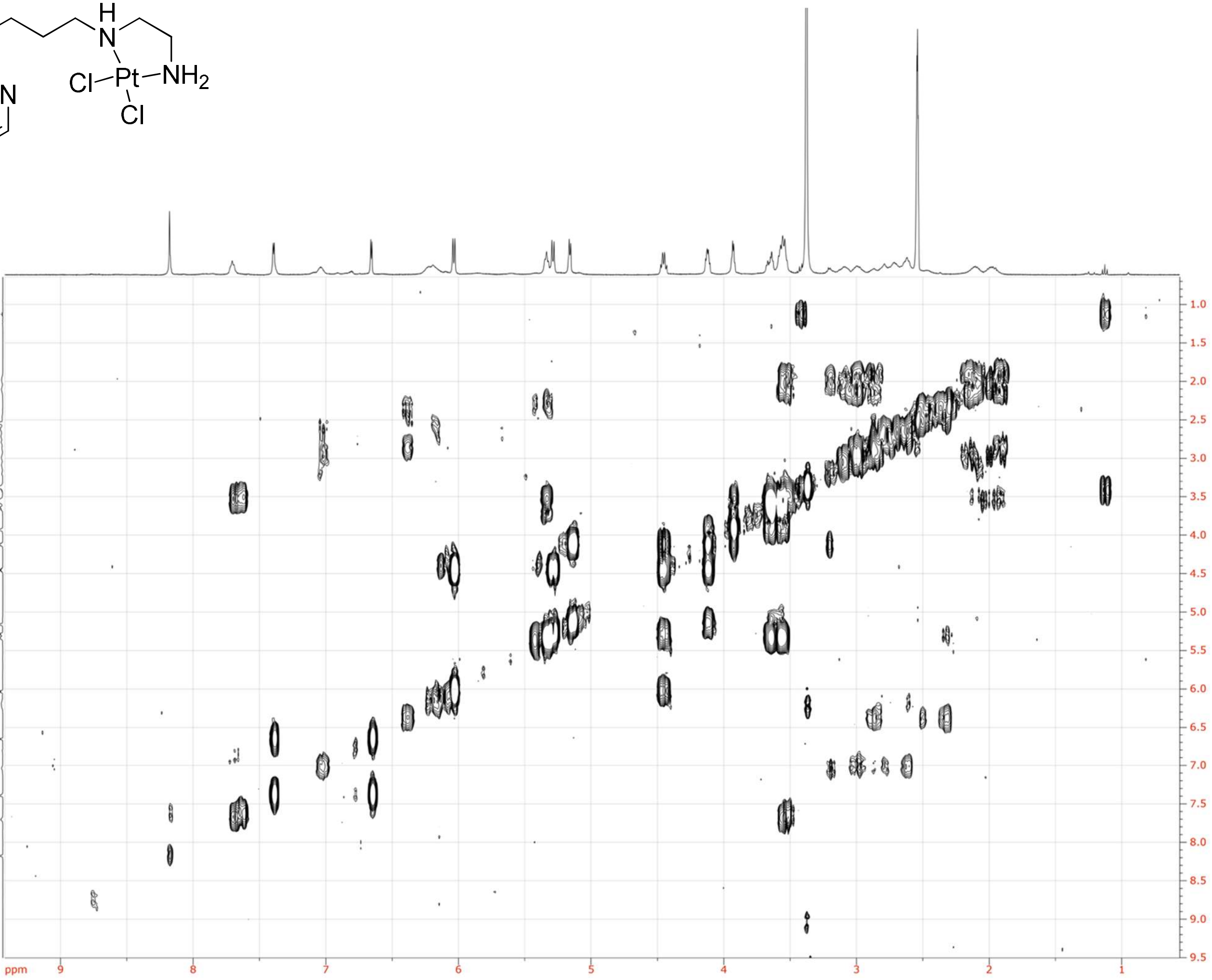

Figure S12. COSY HH spectrum (400 MHz, DMSO-d_6) of complex **6.**

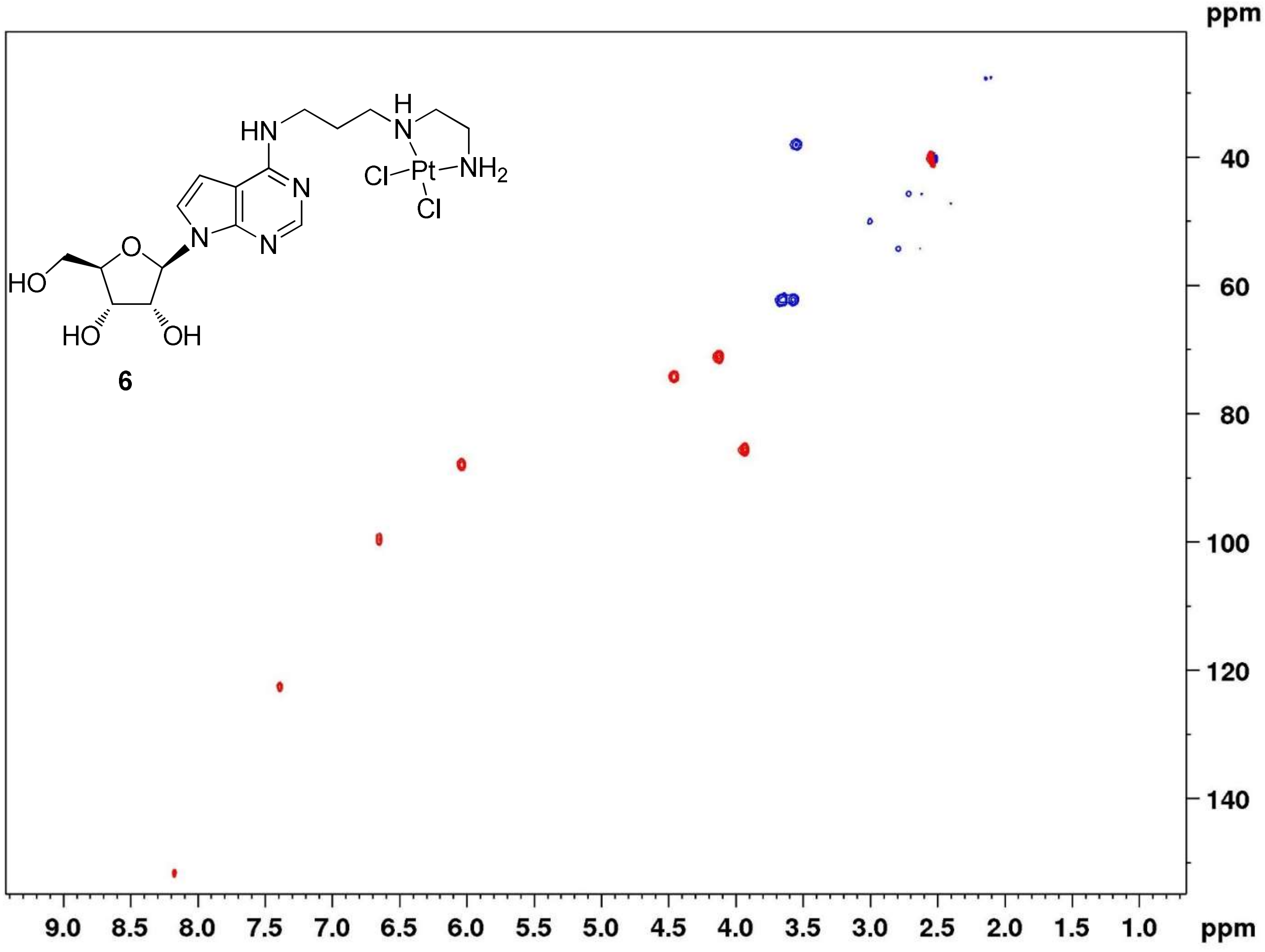

Figure S13. HSQC spectrum (100 MHz, CD_3OD) of complex **6.**

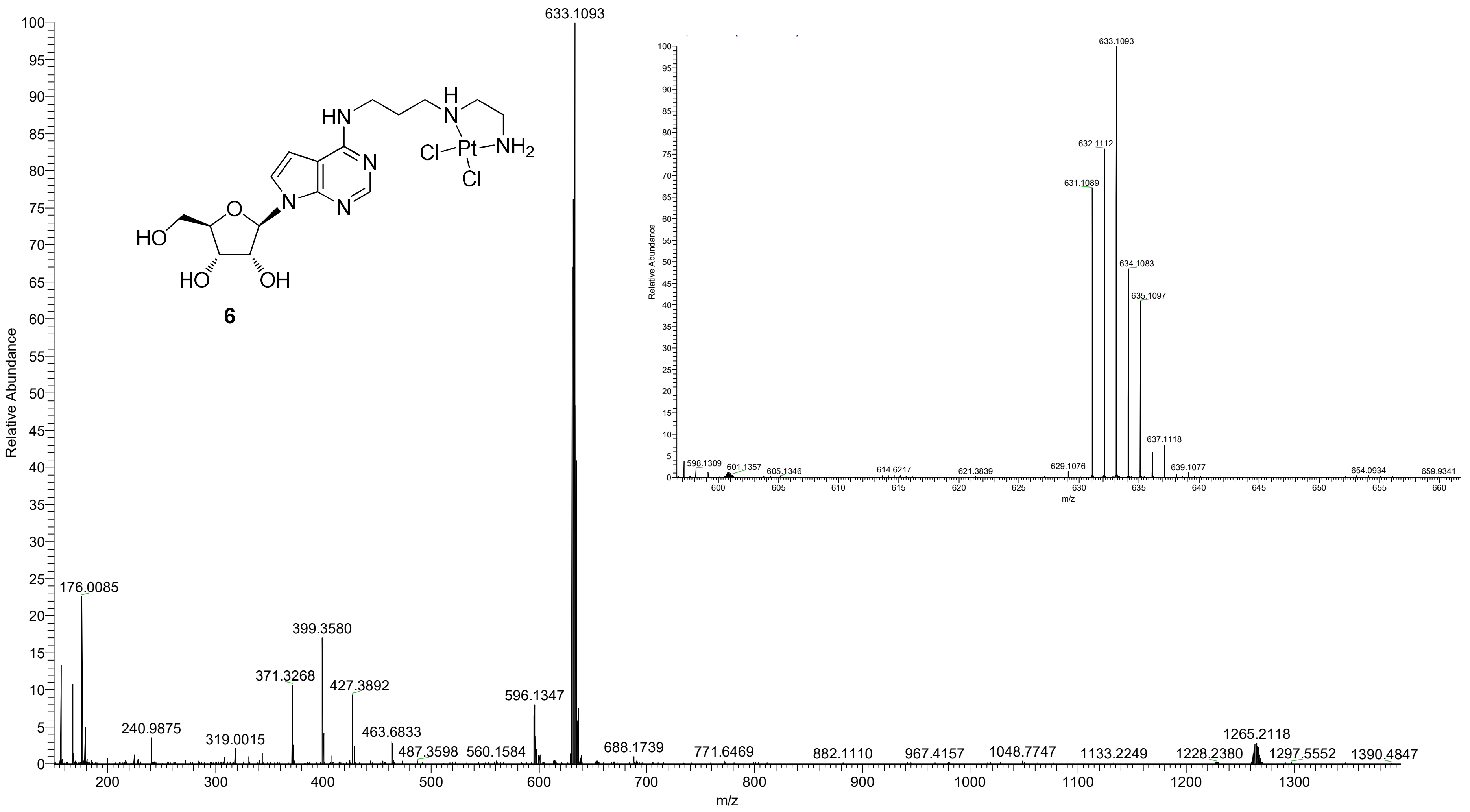

Figure S14. HR ESI MS(+) spectrum of complex **6.**

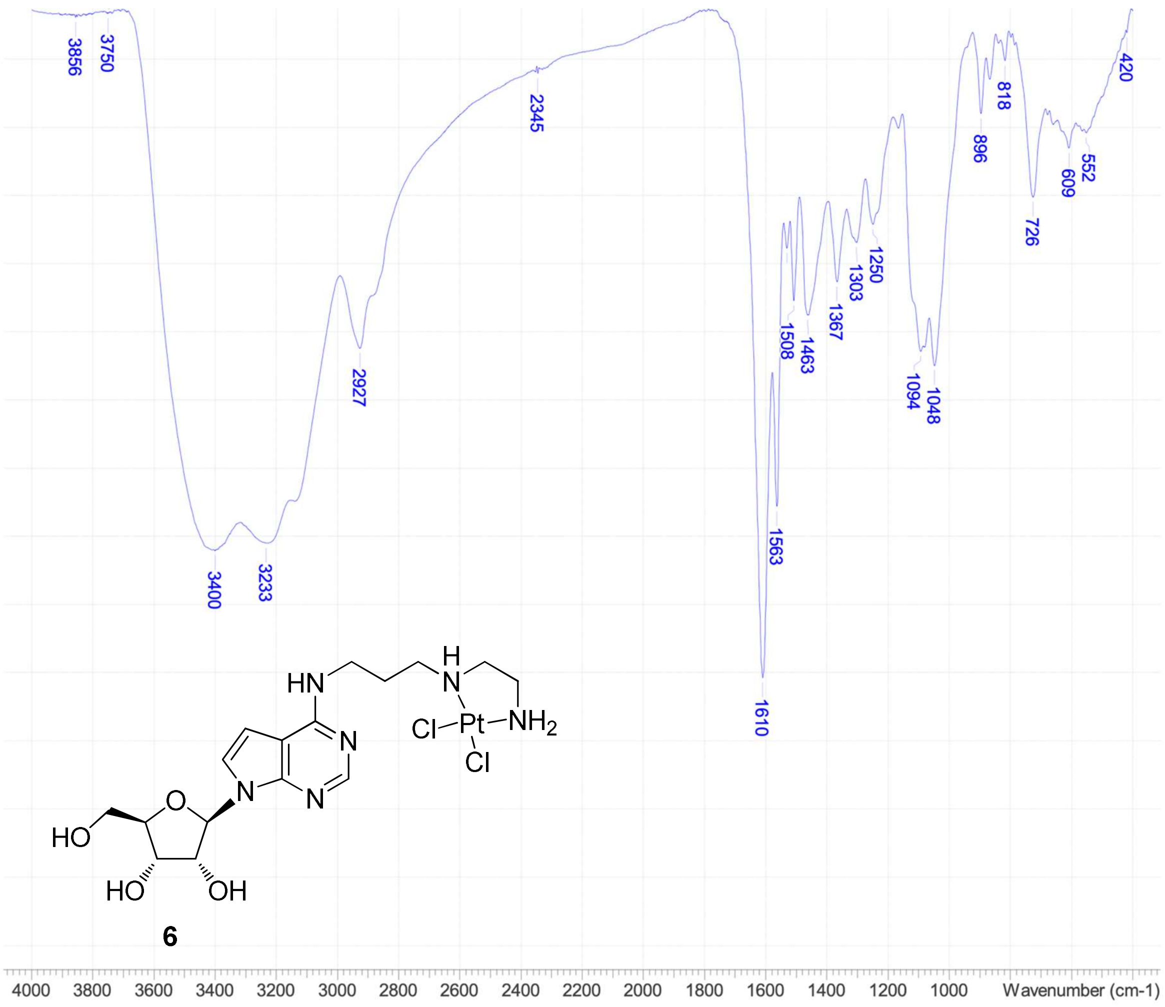

Figure S15. FT IR spectrum of complex **6.**

ODN	*m*/*z*				
		**−3**	**−4**	**−5**	**−6**
**15**	**calcd**	1247.2	935.1	747.9	623.1
	**found**	1246.7	934.8	748.1	623.3
**16**	**calcd**	1181.8	886.1	708.6	590.4
	**found**	1181.3	885.8	708.8	590.4

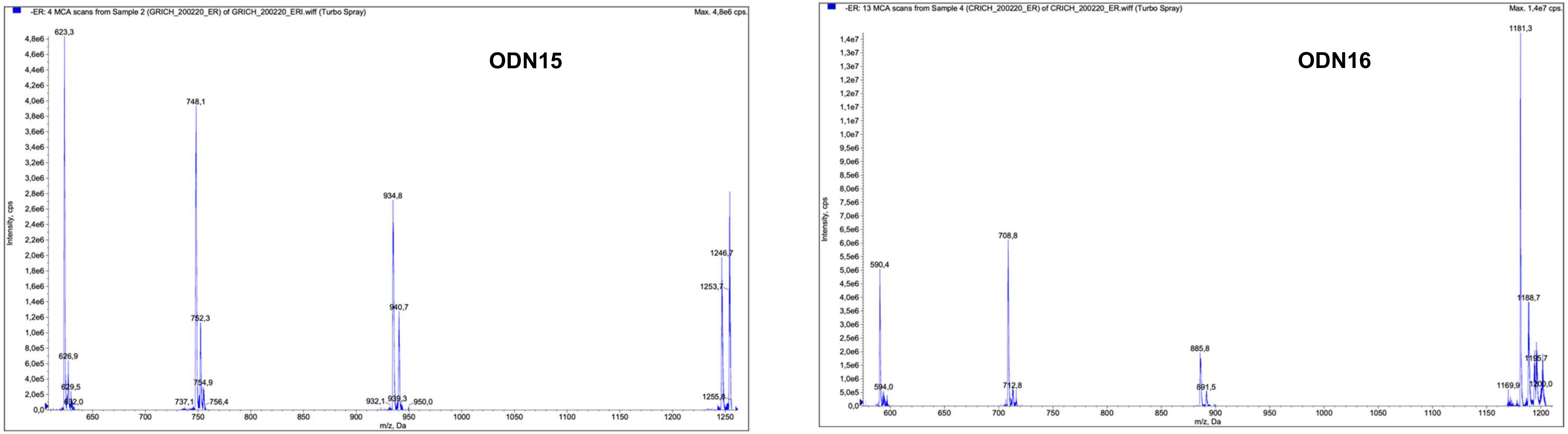

Figure S16. ESI MS(−) spectra of ODNs **15** and **16.**

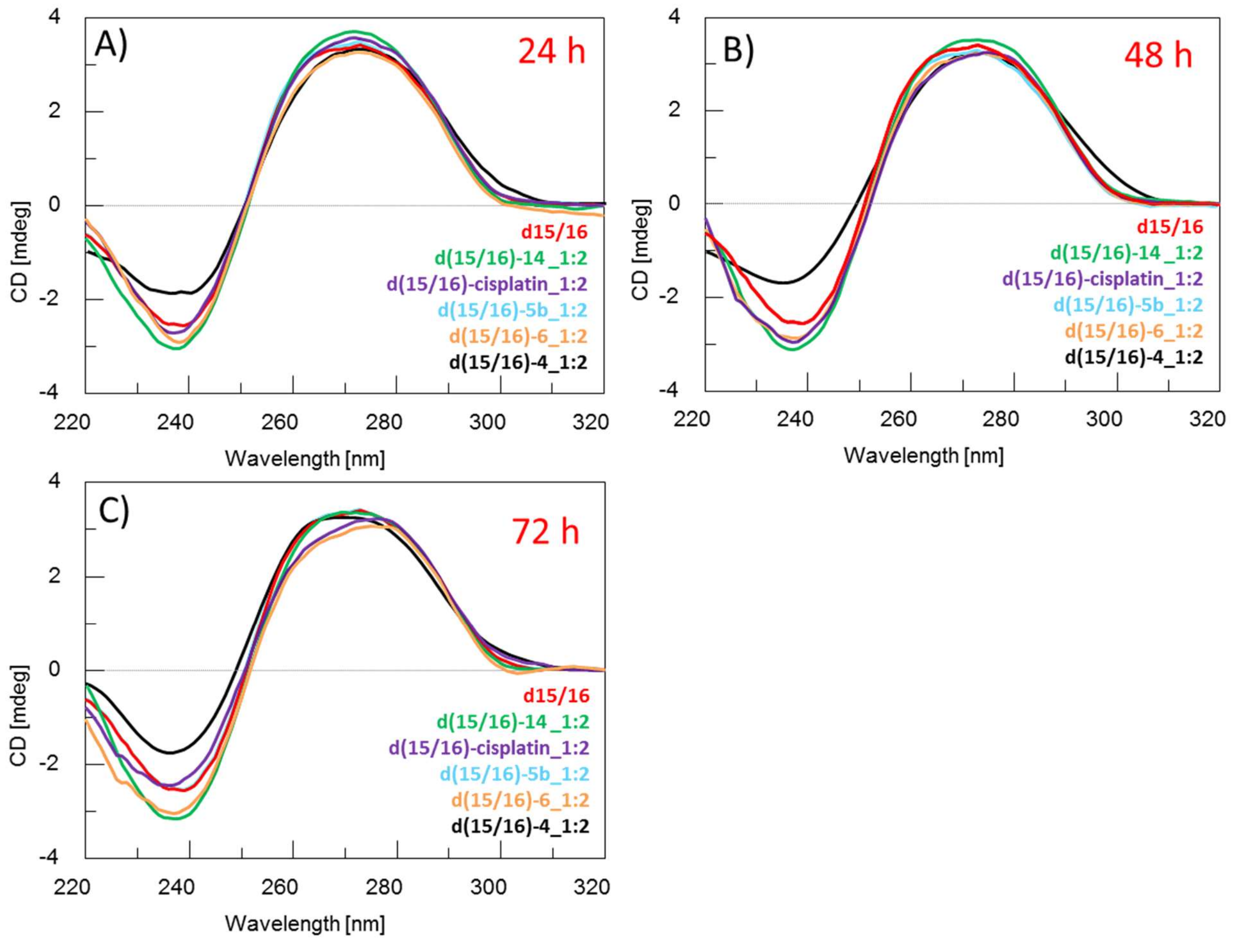

Figure S17. Overlapped CD spectra of **d15/16** recorded at 2 μM concentration (red line) before and after 24 h (panel A), 48 h (panel B) and 72 h (panel C) of incubation with 2 equiv. of **14** (green line), cisplatin (violet line), **5b** (light blue line), **6** (orange line) and **4** (black line) complexes at 37°C. All samples were dissolved in 10 mM PBS/50 mM NaCl-containing buffer at pH 7.0 and the spectra were normalized at 320 nm.

Table S1. The effects on CD profile of the duplex **d15/16** after 24h incubation with 10 equiv. of complex **6**, complex **5b**, cisplatin, diamine **14** and tubercidin **4**. ***T1/2**: Melting Temperature value (λ = 273 nm, 1.0 °C/min heating rate).

**24h**	**Positive band d15/16 λ_{max}/CD (mdeg)/ΔCD**	**Positive band λ_{max}(nm)/CD (mdeg)**	**shift (nm) positive band**	**Negative band λ_{max}(nm)/CD (mdeg)**	**shift (nm) negative band**	**$T_{1/2}$* (°C)**
**d15/16**	273/3.20	273/3.20	-	239/-2.87	-	54
**d(15/16)-6**	273/2.06/1.14	278/2.19	+5	234/-1.34	–5	-
**d(15/16)-cisplatin**	273/2.93/0.27	275/2.99	+2	237/-2.20	–2	-
**d(15/16)-5b**	273/3.00/0.20	274/3.01	+1	235/-0.60	–4	-
**d(15/16)-14**	273/3.78/-0.58	271/3.85	-2	239/-2.75	-	-
**d(15/16)-4**	273/3.76/0.56	273/3.76	-	236/-1.73	–3	55

Table S2. The effects on CD profile of the duplex **d15/16** after 48h incubation with 10 equiv. of complex **6**, complex **5b**, cisplatin, and diamine **14** and tubercidin **4**. ***T1/2**: Melting Temperature value (λ = 273 nm, 1.0 °C/min heating rate).

**48h**	**Positive band d15/16 λ_{max}/CD (mdeg)/ΔCD**	**Positive band λ_{max}(nm)/CD (mdeg)**	**shift (nm) positive band**	**Negative band λ_{max}(nm)/CD (mdeg)**	**shift (nm) negative band**	**$T_{1/2}$* (°C)**
**d15/16**	273/3.20	273/3.20	-	239/-2.87	-	54
**d(15/16)-6**	273/1.62/1.58	281/1.90	+8	228/-3.39	–11	-
**d(15/16)-cisplatin**	273/2.35/0.85	277/2.51	+4	229/-2.09	–10	-
**d(15/16)-5b**	273/1.78/1.42	277/1.85	+4	238/-1.48	–1	-
**d(15/16)-14**	273/3.51/-0.31	272/3.52	–1	238/-3.72	–1	-
**d(15/16)-4**	273/3.43/0.23	273/3.43	-	236/-2.18	–3	55

Table S3. The effects on CD profile of the duplex **d15/16** after 72h incubation with 10 equiv. of complex **6**, complex **5b**, cisplatin and diamine **14** and tubercidin **4**. ***$T_{1/2}$**: Melting Temperature value (λ = 273 nm, 1.0 °C/min heating rate).

**72h**	**Positive band d15/16 λ_{max}/CD (mdeg)/ΔCD**	**Positive band λ_{max}(nm)/CD (mdeg)**	**shift (nm) positive band**	**Negative band λ_{max}(nm)/CD (mdeg)**	**shift (nm) negative band**	**$T_{1/2}$* (°C)**
**d15/16**	273/3.20	273/3.20	-	239/-2.87	-	54
**d(15/16)-6**	273/1.94/1.26	277/2.13	+4	234/-1.81	–5	-
**d(15/16)-cisplatin**	273/1.89/1.31	279/2.15	+6	232/-2.02	–7	-
**d(15/16)-5b**	272/2.08/1.12	276/2.12	+3	239/-1.25	-	-
**d(15/16)-14**	272/3.46/-0.25	273/3.45	-	237/-3.85	–2	52
**d(15/16)-4**	273/3.08/-0.12	273/3.08	-	238/-2.18	–1	55

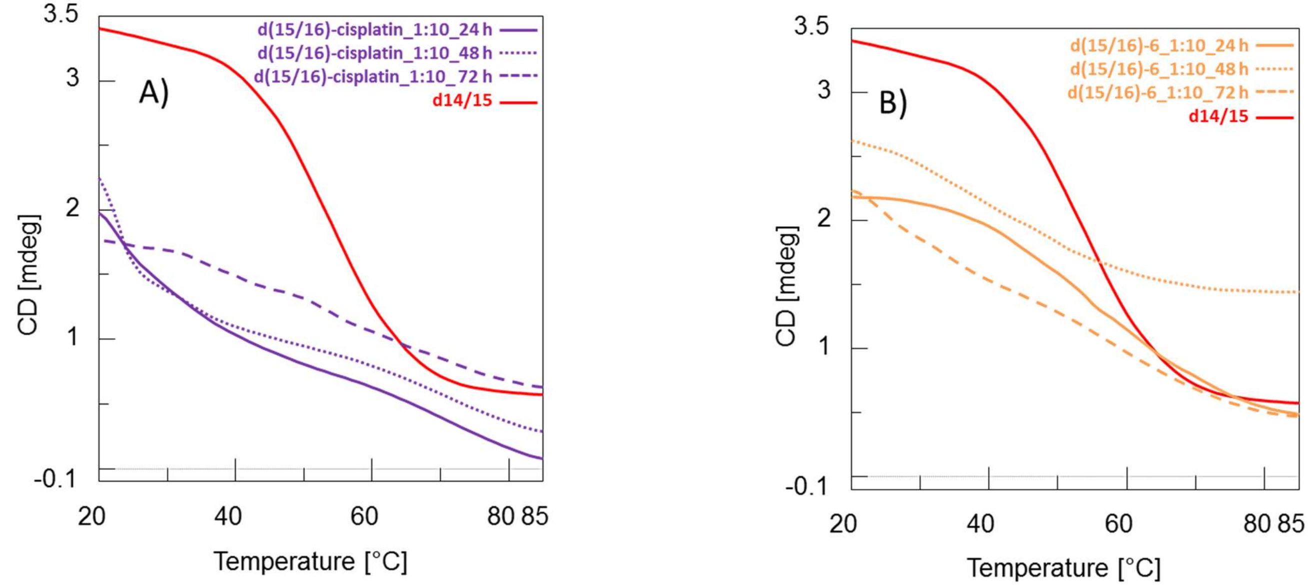

Figure S18. Panel A: overlapped CD melting spectra of **d15/16** recorded at 2 μM concentration (red line) before and after 24h (violet solid line), 48 (violet dotted line) and 72h (violet dashed line) of incubation with 10 equiv. of cisplatin from 20 to 85 °C. All samples were dissolved in 10 mM PBS/50 mM NaCl-containing buffer at pH 7.0 and the spectra were normalized at 320 nm; Panel B: overlapped CD melting spectra of **d15/16** recorded at 2 μM concentration (red line) before and after 24h (orange solid line), 48 (orange dotted line) and 72h (orange dashed line) of incubation with 10 equiv. of complex **6** from 20 to 85 °C. All samples were dissolved in 10 mM PBS/50 mM NaCl-containing buffer at pH 7.0.

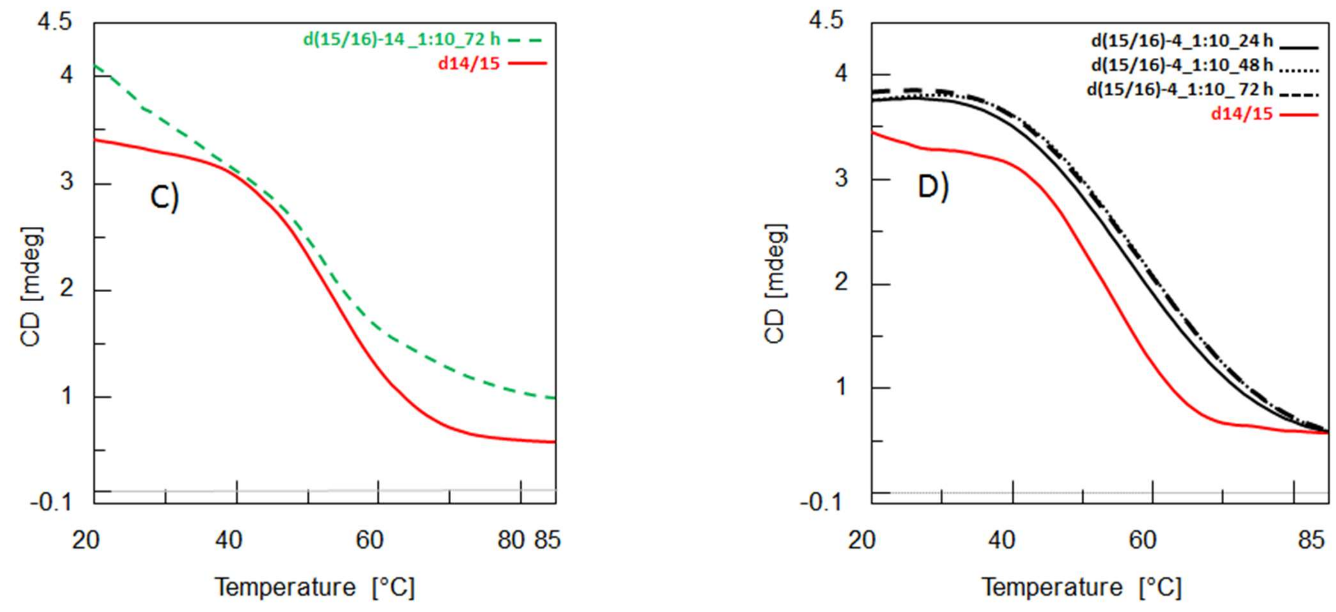

Figure S19. Panel C: overlapped CD melting spectra of **d15/16** recorded at 2 μM concentration (red line) before and after 72h (green dashed line), of incubation with 10 equiv. of compound **14** from 20 to 85 °C. All samples were dissolved in 10 mM PBS/50 mM NaCl-containing buffer at pH 7.0 and the spectra were normalized at 320 nm; Panel D: overlapped CD melting spectra of **d15/16** recorded at 2 μM concentration (red line) before and after 24h (black solid line), 48 (black dotted line) and 72h (black dashed line) of incubation with 10 equiv. of complex **4** from 20 to 85 °C. All samples were dissolved in 10 mM PBS/50 mM NaCl-containing buffer at pH 7.0.

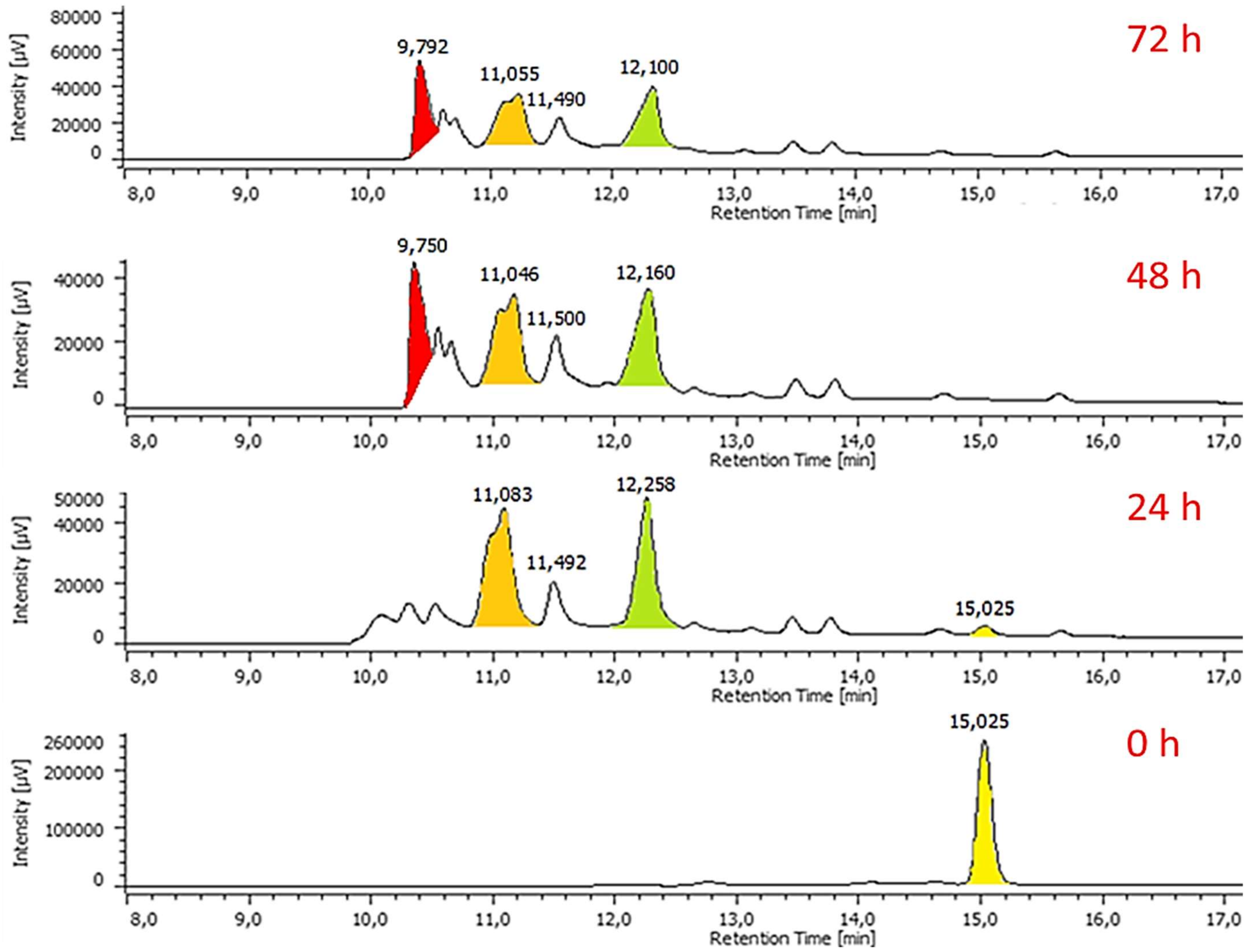

Figure S20. The stability of the complex **6** (0.5 mM in a 0.9% NaCl solution containing 0.5% DMSO) was monitored at 24, 48 and 72h by HPLC. The UV detector was set at 260 nm.

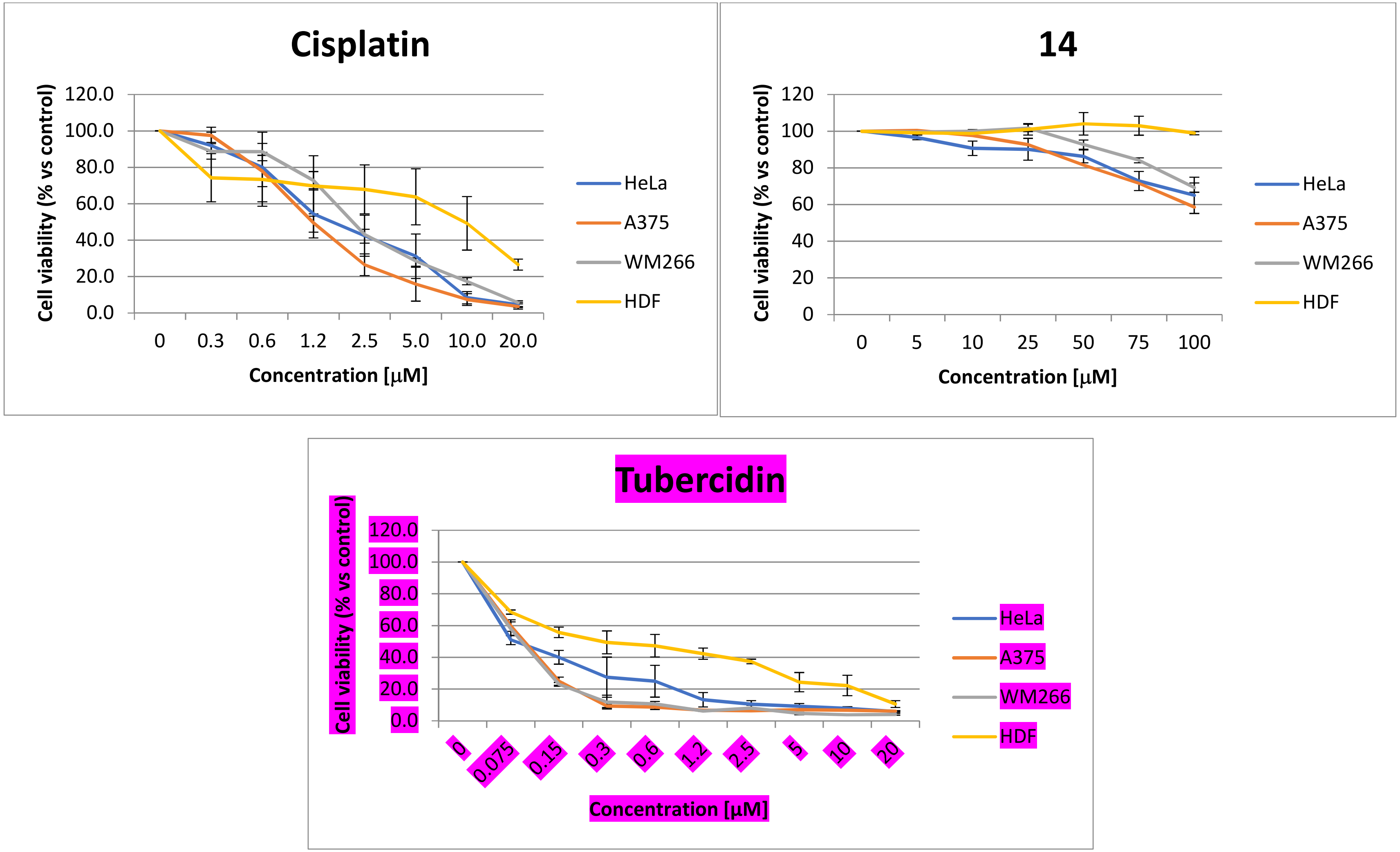

Figure S21. Cell viability assays of cisplatin, tubercidin and diamine **14** on HeLa, A375, WM266, and HDF. The cells were incubated in the presence of cisplatin, tubercidin and the diamine **14** at the indicated concentrations at 37 °C for 72h. Cell viability was measured by using MTT assay. The results are presented as the percentage of proliferating cells respect to the control (vehicle treated cells) and are expressed as means ± SE of at least three independent experiments.

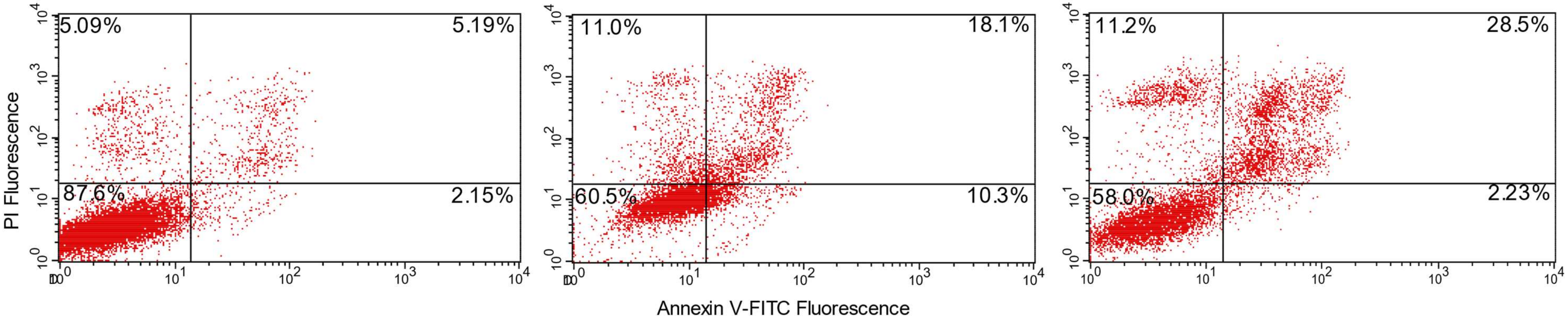

(**A**) (**B**) (**C**)

Figure S22. Apoptosis analysis with annexin V-FITC and PI double staining on HeLa cells. HeLa cells were seeded at 40.000 cells/well on 6-well plate and treated with IC_{50} concentration of cisplatin or tubercidin for 72h. Panel A: control (vehicle treated cells); Panel B: cells treated with 1.8 μM cisplatin; Panel C: cells treated with 0.11 μM tubercidin. Lower left quadrant: viable cells; upper left: necrotic cells; upper right: advanced apoptotic cells; low right: early apoptotic cells.
